# Selection for ancient periodic motifs that do not impart DNA bending

**DOI:** 10.1371/journal.pgen.1009042

**Published:** 2020-10-06

**Authors:** Aletheia Atzinger, Jeffrey G. Lawrence

**Affiliations:** University of Pittsburgh, Department of Biological Sciences, Pittsburgh, United States of America; Uppsala University, SWEDEN

## Abstract

A ~10–11 bp periodicity in dinucleotides imparting DNA bending, with shorter periods found in organisms with positively-supercoiled DNA and longer periods found in organisms with negatively-supercoiled DNA, was previously suggested to assist in DNA compaction. However, when measured with more robust methods, variation in the observed periods between organisms with different growth temperatures is not consistent with that hypothesis. We demonstrate that dinucleotide periodicity does not arise solely by mutational biases but is under selection. We found variation between genomes in both the period and the suite of dinucleotides that are periodic. Whereas organisms with similar growth temperatures have highly variable periods, differences in periods increase with phylogenetic distance between organisms. In addition, while the suites of dinucleotides under selection for periodicity become more dissimilar among more distantly-related organisms, there is a core set of dinucleotides that are strongly periodic among genomes in all domains of life. Notably, this core set of periodic motifs are not involved in DNA bending. These data indicate that dinucleotide periodicity is an ancient genomic architecture which may play a role in shaping the evolution of genes and genomes.

## Author summary

Chromosomes are DNA polymers comprising hundreds of millions of instances of 4 different nucleotide building blocks. Chromosomes are often viewed as repositories of genes, which are small DNA regions encoding products which cooperate to make an organism. But chromosomes are also enormous, unwieldy molecules that must be maneuvered in various ways. Due to their bulk, maneuverability would be greatly facilitated by information embedded within and between genes which assists those processes. This infrastructure would enable cells to manipulate genomes efficiently; disrupting it would be disastrous. We examined the spacings between all possible pairs of nucleotides (dinucleotides) and found that different dinucleotides were strongly periodic in different organisms. That is, certain spacings were more frequently observed, and were integral multiples of a single spacing (the period). The period varied among organisms between 9.7 and 11.8 nucleotides; contrary to long-held assumptions, the period was not related to organismal growth temperature. More closely-related organisms were more likely to have similar periods and share which dinucleotides were periodic, but even distantly-related organisms shared a core of periodic dinucleotides. This ancient periodicity likely assists cells in maneuvering the vast DNA polymer, and differences between organisms could restrict the flow of genes between them.

## Introduction

A dominant mode of bacterial genome evolution is the acquisition of foreign DNA via lateral gene transfer, or LGT [1, 2]. While bacteria are capable of acquiring large amounts of DNA from many donors [1, 3], most incoming fragments are lost [4, 5]. Many acquired regions confer benefits–such as antibiotic resistance or the ability to degrade a novel food source–which increase their likelihood of long-term retention. However, as foreign DNA may also incur a detriment upon insertion into a new recipient genome, the acquisition must have a net positive impact on fitness to be successful [6–9]. In addition to being a repository of information, a chromosome is a large polymer that must be maneuvered in various ways, such as compaction [10], segregation during cell division [11], or accessibility of genes during transcription [12]. These processes would be facilitated by information embedded within and between genes, termed genomic architecture. Non-native DNA would be detrimental if it interfered with this architecture, and thus interfered with these processes. An acquisition will be retained only if the benefits outweigh these detriments [6–9].

Far from being an abstract concept, such genomic architecture has been demonstrated to exist [8]. Architecture IMparting Sequences, or AIMS, are strand-biased octamers that increase in both abundance and strand polarity with proximity to the replication terminus [8]. Some AIMS aid in DNA translocation during cell division by providing the sites for loading the FtsK DNA translocase, which pumps DNA across the septum of the dividing cell [13]. The distribution and conservation of AIMS shows that they are under selection [9]. In addition, the distribution of inversions within the genome–they are both smaller and less abundant near replication termini–is consistent with their counterselection when they disrupt AIMS [9]. AIMS vary in sequence identity between different taxonomic groups; they are shared among closely related organisms, and less so in more distantly related ones [8]. Critically, they are strand-biased even within recently acquired insertions [9], indicating that insertions with non-permissive AIMS were lost by deletion. That is, insertions which disrupted this architecture would have incurred a detriment, and were thus removed from the chromosome.

An additional genomic architecture may be the consistent spacing between iterations of a specific nucleotide motif (genomic periodicity). A ~10.5 bp periodicity in the distribution of AA/TT dinucleotides has been observed in eukaryotes [14]. The base-stacking of this dinucleotide induces a bend in the DNA [15], and this periodicity was found to coincide with nuclease protection afforded by histone binding [16]. Because relaxed B-DNA has helical pitch of 10.5 bp [17], periodic bending could facilitate DNA wrapping around the histone core. While AA/TT dinucleotides induce a bend, all WW dinucleotides–W(weak) being A or T–are more flexible and can be more readily bent than other dinucleotides [18].

A similar periodicity of WW dinucleotides was observed in mesophilic Bacteria (~11.0 bp period) and in thermophilic Archaea (~10.0 bp period) [19]. The difference in periods was attributed to the negative or positive DNA supercoiling characteristic of low and high temperature prokaryotes, respectively. Organisms growing at lower temperatures (< 60 C) have negatively-supercoiled DNA, which lowers the melting point of duplex DNA to allow for efficient transcription [20]. Organisms growing at higher temperatures (> 60 C) have more positively-supercoiled DNA, which is instrumental in preventing the melting of dsDNA at those temperatures [21]. The suggestion made by Herzel and colleagues was that the periodicity of WW dinucleotides reflects its universal role in DNA folding (*e*.*g*., bending around compaction proteins), and that variation in the period reflects differences in organismal growth temperatures [19].

However, further work with a larger number of genomes suggested that the spectrum of observed periods among WW dinucleotides may be more varied and, more importantly, not determined solely by organismal growth temperature [22]. However, all estimates of the period by Mrázek were made using a Fourier analysis, which lacks accuracy due to blurring of the Dirac function by edge effects [23]. Moreover, the lack of variance estimates for genomic periods precludes a robust test of this hypothesis. Yet, if his conclusions are true, it raises the question of whether periodicity in WW, or any other dinucleotides, is driven by selection for DNA bending. Moreover, if different dinucleotides are periodic within different phylogenetic groups, then dinucleotide periodicity may impart a genomic architecture that could constrain LGT between them. To explore this, we developed more robust methods to assess the periodicity of all dinucleotides, to determine which motifs were periodic, to verify that selection was responsible for these distributions, and to compare genomic periodicity between genomes. Our results suggest that selection for periodicity does not arise from its role in DNA bending, and that variability in periodicity is sufficient to constraint LGT between distantly-related taxa.

## Results

### Periodicity is measured robustly

**Fig 1:**
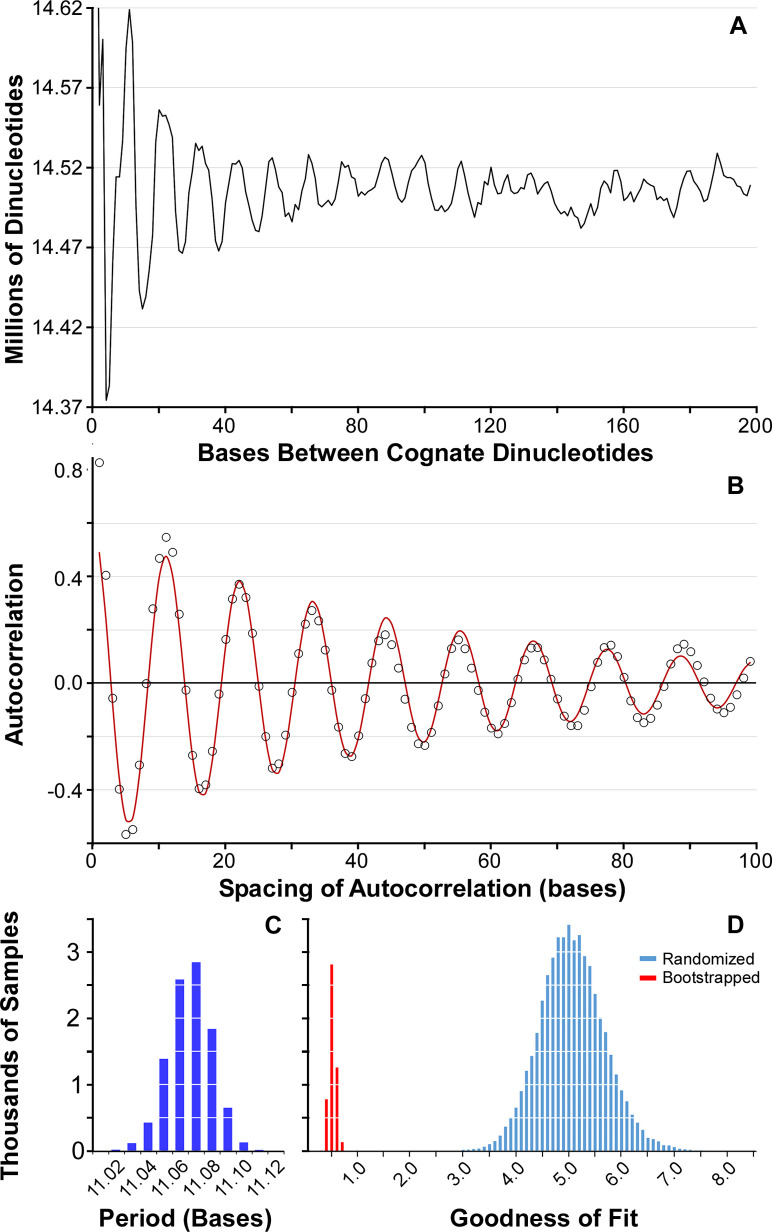
Periodicity can be measured robustly. (A) The abundances of collated dinucleotide spacings per interval in *E*. *coli* K12 DH10b. (B) Autocorrelation of the abundances of spacings in A, with a damped sine curve fit to these data. (C) Abundances of genomic periods obtained for 10,000 bootstrap samplings of dinucleotide spacings in *E*. *coli* K12 DH10b. (D) Goodnesses of fit for damped sine curves fit to autocorrelation data when randomized with respect to the intervals are shown in blue. The goodnesses of fit for the 10,000 bootstrapped resamples from Fig 1C are shown in red.

We measured and collated spacings between 100 degenerate and nondegenerate dinucleotides (see methods) in the *Escherichia coli* genome. There is an apparent enrichment for spacings at integral multiples of ~11 bp, with a ~2 percent variation in abundance across different spacings (Fig 1A). To reduce noise, we performed an autocorrelation on the abundances of spacings before fitting a damped sine curve, with a goodness of fit (GdF) of 0.539, to obtain an estimate of the period as 11.07 bp (Fig 1B). We specify the genomic period as the period estimated from all dinucleotides in a genome. To obtain a confidence interval for the genomic period, we performed 10,000 bootstrap resamples of the spacings (Fig 1C); the distribution of resampled periods was Gaussian with a range between 11.03 and 11.11 bp, a mean of 11.066 bp, and a standard deviation of 0.013 bp. This small variance is consistent with the very small deviation of observed autocorrelation values from the optimally-fitted curve and predictive of a small goodness of fit (S1 Fig). In contrast, an artificial *E*. *coli* genome maintaining the codon-position-specific dinucleotide, trinucleotide and tetranucleotide frequencies, gene length distribution, and strand bias of the genuine *E*. *coli* genome shows no evident periodicity (period = 6.18 +/- 4.23 bp; GdF = 5.15; S2A Fig).

While the fit of the curve in Fig 1B is visually impressive, even nonperiodic data can be fit (albeit poorly) to a damped sine curve (see S2A Fig). To test whether the fit is meaningful, we randomized the autocorrelations with respect their spacing 10,000 times, each time fitting a curve. From this we obtained a distribution of GdFs that would be seen for non-periodic data (blue bars in Fig 1D; μ = 5.11, σ = 0.61). From this distribution, the probability of observing a GdF of 0.539 is P = 3.56x10^-14^; the GdFs for the 10,000 bootstrapped replicates are shown in red in Fig 1D (μ = 0.565, σ = 0.062). As demonstrated above (S2A Fig), the artificial *E*. *coli* genome has a fit no better than would be seen at random. Therefore, we conclude that dinucleotides are significantly periodic within the *E*. *coli* genome, and that we have measured its period to within ~0.05 bp.

To determine if this distribution of random GdF values is generalizable across genomes, and thus allows us to infer the significance of periodicity from the goodness of fit to genuine data without the need to randomize the autocorrelations every time, we repeated this procedure for six taxa, one from each of six different divisions, whose genomes vary in length from 0.4 MB to 6 MB and in %GC content from 20% to 74%. Each distribution of GdF values from randomized spacings was determined to be Gaussian (Kolmogorov-Smirnov test), and their means and variances were not significantly different (S1 Table). From the combined distribution of 70,000 randomizations, the P-value for GdF < 3.0 was 2.7x10^-4^ (S1 Table); this value (GdF < 3.0) is henceforth used as a threshold for robust fits for all analyses.

Although the period measured for *E*. *coli* is 11.07 bp, it is possible that this period is influenced by genes encoding repeated protein motifs, so that the 11.07 bp period reflects a combination of 9- and 12-bp repeated motifs. If protein motifs were responsible for the periodicity we observe, then ignoring spacings between dinucleotides both lying at the first and second codon positions within the same gene should eliminate (or at least dramatically weaken) the periodicity we measure. In contrast, ignoring spacings between dinucleotides both lying at the third and first codon positions within the same gene would not weaken the periodicity. We observe the opposite, whereby ignoring spacings between dinucleotides lying at the first and second codon positions somewhat strengthens periodicity in the 27 genomes measured (S3 Fig); in contrast, ignoring spacings between dinucleotides both lying at third and second codon positions within the same gene somewhat weakens the strength of periodicity. This suggests that any repeated motifs in protein-coding sequences actually detract from the genomic period. Therefore, we conclude that the periodicity we observe is not imparted by repeated protein motifs.

### Genomic period is not predicted by organismal growth temperature

**Fig 2:**
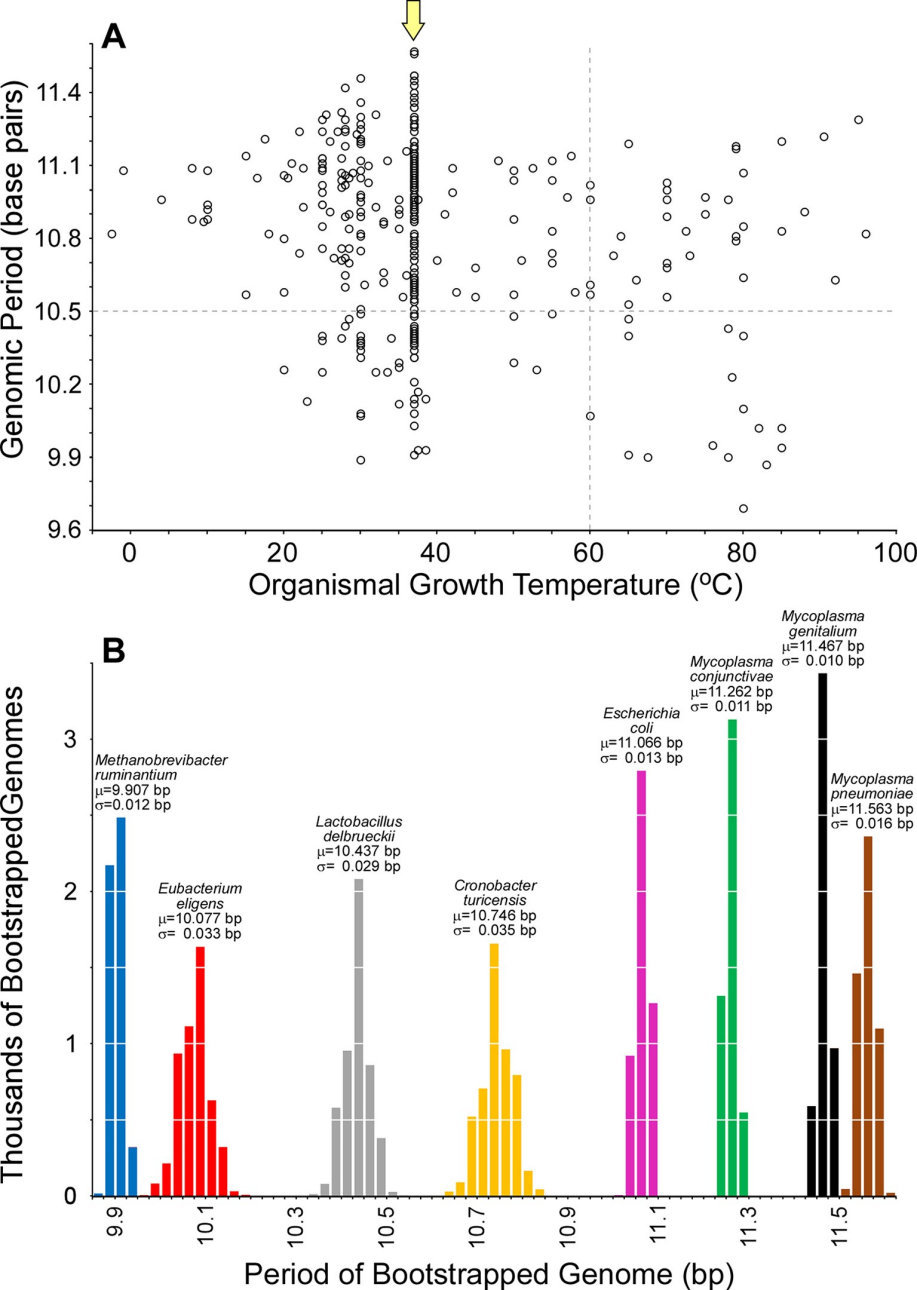
The genomic period is not strongly correlated to organismal growth temperature. (A) The periods of 358 genomes are plotted against the organismal growth temperatures. The temperature above which genomes are positively supercoiled is indicated by a vertical dashed line, and the helical pitch of relaxed B-DNA is shown as a horizontal dashed line. A yellow arrow indicates organisms growing at 37°C. (B) Bootstrapped periods for the genomes of eight organisms growing at 37°C.

Herzel and colleagues [19] proposed that the period of WW dinucleotides reflects supercoiling direction. Such periodic bending would assist DNA wrapping around a cylinder (*e*.*g*., histones or histone-like proteins). If this were the primary function, then the period would be expected to be different in organisms with strongly positively- or negatively-supercoiled DNA. Organisms growing at or above 60°C (vertical dashed line in Fig 2A) should have positively supercoiled genomes, with the degree of positive supercoiling increasing with increased temperature [21]; if periodicity assists in wrapping DNA around a cylinder, one would expected increasingly smaller periods (less than 10.5 bp, horizontal dashed line in Fig 2A) with increasing temperature. In contrast, organisms growing below 60°C should have negatively supercoiled genomes, with the degree of negative supercoiling increasing with decreased temperature; one would expect increasingly longer periods with decreasing temperature in these organisms.

To test whether the genomic period is robustly predicted by the direction and degree of supercoiling, we analyzed the genomes of 358 organisms with a range of preferred growth temperatures (-2.5°C to 96°C), where growth temperature is a surrogate measure of supercoiling [24]. Genomes were chosen with a maximal 16S rRNA similarity of 97%, ensuring that periods measured in different organisms were estimated from sequences with significant differences; all periods were deemed significant (GdF < 3.0). The data do not show a strong relationship between genomic period and organismal growth temperature (R^2^ = 0.046; Fig 2A), which is contrary to what was expected if the period were predicted by the direction and magnitude of supercoiling. The majority of genomes growing at or above 60°C have genomic periods greater than 10.5 bp, and many mesophilic organisms have genomic periods less than 10.5 bp (Fig 2A). This suggests that the function of periodic sequences is not to assist in periodic bending around a cylinder.

To determine if these periods have been measured robustly, we estimated the variance in genomic periods for 8 organisms all growing at 37°C. If genomic period is strongly predicted by growth temperature, these genomes should show similar periods, where our varied estimates could have resulted from large degrees of error. However, our estimates of genomic periods are robust; mean periods for the 8 genomes range from 9.93 bp to 11.56 bp, with standard deviations ranging from 0.012 to 0.035 bp. Despite all growing at 37°C (well below the 60°C threshold for organisms with positively supercoiled DNA), 3 of the 8 genomes have robustly measured periods less than 10.5 bp (Fig 2B). While other factors can influence the degree of supercoiling, it is implausible that bacteria growing at 37°C have positively supercoiled genomes with a ~10 bp helical pitch. This lack of strong correlation between period and organismal growth temperature is still evident when using the period of only WW dinucleotides (R^2^ = 0.126). Therefore, we conclude that the period is not strongly predicted by the magnitude or direction of supercoiling.

### Periodicity is under selection

One explanation for robust periods which cannot be robustly predicted by the magnitude or direction of supercoiling is that observed periodicity in dinucleotide abundances reflects mutational biases and serves no functional role. If so, then such periodicity would be most evident in genomes where selection for other information did not act to overwrite those patterns. For example, selection for amino acid choice would obscure mutational biases. Genomes with low ratios of change at nonsynonymous sites (K_A_) to synonymous sites (K_S_) experience strong selection for amino-acid conservation in the face of underlying mutational biases. If periodicity were solely imparted by mutational biases, it should be stronger in genomes with high K_A_/K_S_ ratios and become weaker in genomes with low K_A_/K_S_ ratios, where selection for amino-acid choice would eliminate the underlying mutational bias. In contrast, if periodicity is the result of selection, then it would be stronger in genomes with low K_A_/K_S_ ratios, where selection also acts to favor preferred amino acids.

**Fig 3:**
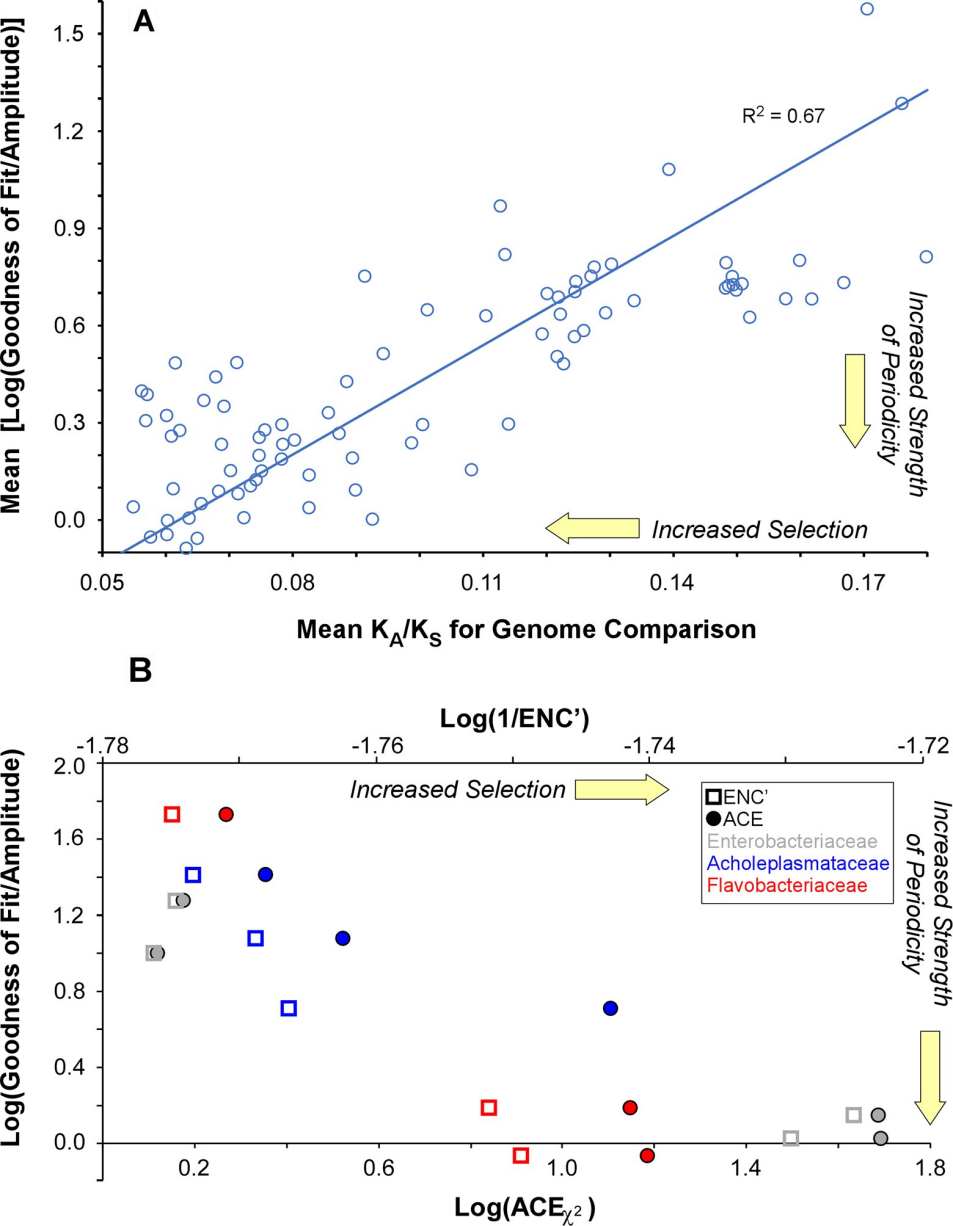
Periodicity is maintained by selection. (A) Mean strengths of periodicity for pairs of organisms in the same genus are plotted against the K_A_/K_S_ ratio between each pair. (B) Strength of periodicity for genomes of Bacteria in three different families from three different Divisions is plotted against two metrics of codon bias (ACEχ^2^ and ENC’ [25, 26]). For metrics of codon selection, genomes of Enterobacteriaceae (γ-proteobacteria) used 182 genes, those of Acholeplasmataceae (Tenericutes) used 264 genes, and genomes of Flavobacteriaceae (Bacteroidetes) used 169 genes.

To test this hypothesis, we measured the divergence at synonymous and nonsynonymous sites in 84 pairs of genomes across 25 families in 11 divisions of bacteria. Genome pairs were chosen with K_S_ values between ~0.3 and ~1.9, allowing robust measurement of K_A_/K_S_ ratios, and all genomes had significant periodicity (GdF < 3.0). As shown in Fig 3A, the average strength of periodicity in each genome pair increased as the strength of selection increased. This suggests that periodicity is not imparted solely by mutational biases but instead is information under selection that is more evident when the strength of selection increases.

If so, we predict that genomes under the weakest selection would show little evidence of periodicity. Codon selection favors particular codons in moderately and highly expressed genes, and would also act to obscure any periodic abundances of dinucleotides generated by mutational biases. As a result, if it were solely imparted by mutational biases, genomic periodicity should be more evident in genomes lacking codon selection. In contrast, if periodicity in dinucleotide abundance serves a function and is maintained by selection, then it would be absent in genomes lacking strong codon selection. Codon selection is weakest in genomes of endosymbiotic bacteria. We measured the strength of codon selection in 10 genomes from 3 families of bacteria using two metrics of codon selection, ACE- χ^2^ and ENC’ [25, 26]. Codon selection is minimal or undetectable in obligate endosymbionts, whereas strong codon selection is observed in fast-growing, free-living bacteria [27].

We compared strength of periodicity and strength of selection for both known obligate endosymbionts and fast-growing, free-living bacteria within three bacterial families drawn from three different divisions. For genomes in the same family, we assessed codon selection in the subset of genes with orthologues in all taxa analyzed in that family, allowing comparison of strength of codon selection between organisms. To accommodate for differences in genome size in calculating strength of periodicity, the numbers of dinucleotide spacings used to calculate autocorrelations in each genome was set to the number observed in the smallest genome analyzed in in each family. In all clades, stronger periodic signals correlate with stronger codon selection (Fig 3B); this was true even if the strength of periodicity was measured only within genes shared between members of each family. These data confirm the conclusions drawn from assessing strength of selection by amino acid conservation (Fig 3A). Therefore, we conclude that periodicity is under selection and does not result from mutational biases alone.

### Most periodic dinucleotides do not impart the ability to bend

**Fig 4:**
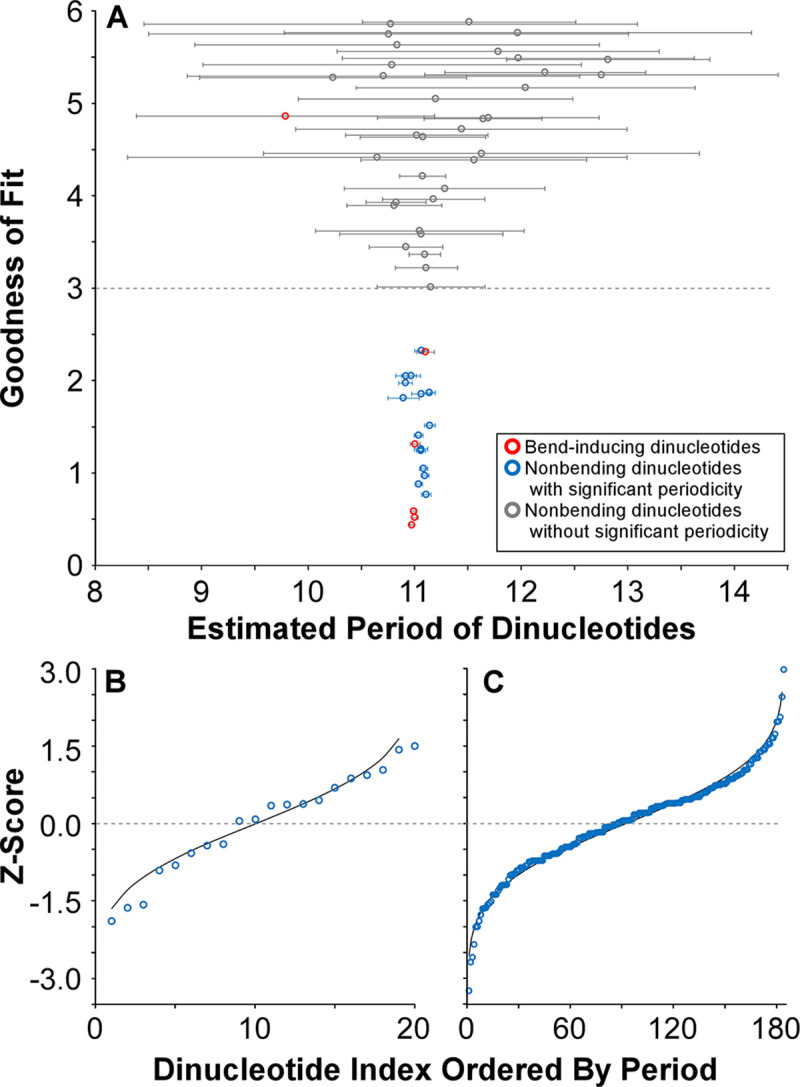
Periodic dinucleotides within a genome share the same period. (A) Bootstrapped period estimates for each dinucleotide in *E*. *coli* K12 DH10b are plotted against the goodness of fit of the fitted curve. Bend-inducing dinucleotides are indicated by red dots; the threshold for significant periodicity (GdF < 3.0) is indicated by a dashed grey line. Error bars for the periods represent two standard deviations of the distributions of periods for 1000 bootstrapped resamples of the dinucleotide spacings. (B) Distribution of variance-normalized periods for significantly periodic dinucleotides (GdF < 3.0) in the *E*. *coli* K12 DH10b genome. (C) Distribution of 184 variance-normalized periods for significantly periodic dinucleotides in the *E*. *coli* K12 DH10b, *Methanothermobacter marburgensis* str. Marburg, *Mycoplasma hyopneumoniae* 232, *Flavobacterium psychrophilum* JIP02/86, and *Synechococcus* sp. CC9311 genomes. Curves in Fig 4B and 4C represent the Gaussian cumulative distribution curves given the means and variances of those distributions.

Since the genomic period is not predicted by magnitude or direction of supercoiling (Fig 2), it is not clear which dinucleotides will be, or should be, periodically distributed. To identify which dinucleotides are periodically distributed within *E*. *coli*, we fit curves to the autocorrelation data for individual dinucleotides, exploring the range of periods within 5 bp of the genomic period (6.07 to 16.07 bp, Fig 4A); spacings for each dinucleotide were collated for the two replicores, and the variance of each period was determined by bootstrapping. Twenty dinucleotides had robustly measurable periods (GdF<3.0; dashed line in Fig 4A), with low variation (error bars in Fig 4A). Among dinucleotides that either induce a bend or confer flexibility (red points in Fig 4A) [15, 18], only 5 were periodic whereas one was not; if periodicity is maintained to promote DNA bending, one would expect all dinucleotides imparting bends to be periodically distributed. Strikingly, the majority (15 of 20) of periodic dinucleotides have no known role in promoting DNA bending: AR, CK, CS, GS, GY, RS, RW, SK, SM, SS, TY, WK, WM, YS, AND YW. Several of these dinucleotides contain no possible A or T bases at all (CS, GS, SS). When periodic dinucleotides are considered individually, none are strongly correlated with growth temperature (mean R^2^ = 0.096). Thus, we conclude that selection for genomic periodicity does not reflect solely a role in DNA bending.

### Periodic dinucleotides appear to show a single, common period

There is variability in the inferred periods for the 20 significantly periodic dinucleotides in *E*. *coli* (Fig 4A). To test whether this variability reflects stochastic error or if there is selection for different functions and, therefore, more than one period in the genome, we performed a Kolmogorov-Smirnov test for normality among robustly-measured periods (Fig 4B). The 20 periods estimated for the individual dinucleotides are normally distributed around the global period; no significant difference could be detected from a theoretical distribution (P>0.1). To verify that there is no evidence for multiple periods in *E*. *coli*, we resampled spacings and re-inferred periods of significantly periodic dinucleotides for 100 iterations (S2 Table). Only one of 23 periodic dinucleotides rejected the genomic period at a P-value of 0.05, no more than expected.

Because the low sample size of periodic dinucleotides may preclude robust testing, we examined the distributions of normalized periods for significantly periodic dinucleotides within 209 genomes within 24 families of bacteria across 9 divisions, wherein at least 10 of the 55 palindromic dinucleotides (see below) were significantly periodic (S3 Table). Only 25 of the 209 genomes were significantly different from Gaussian at P = 0.1; this number is not significantly different from what we would expect (P > 0.2, binomial test). As expected, an aggregate distribution of periods from six exemplar genomes was clearly Gaussian (Fig 4C; P>0.1, KS test). Therefore, we have no evidence that multiple periods are under selection in any genome. Henceforth, all assessments of strength of periodicity of individual dinucleotides are measured using curves fit with genomic period.

The variability of the periods of individual dinucleotides, as well as the lack of connection between motifs involved in DNA bending and selection for periodicity, calls into question the robustness of previously published estimates of genomic periods analyzing only WW dinucleotides and related motifs. We obtained bootstrapped estimates for both the genomic period and the period of WW dinucleotides for 82 genomes with previously published periods for WW dinucleotides [19, 22, 28]. While there was general congruence among all period estimates (S4 Table), lack of congruence largely reflected a lack of meaningful periodic signal (genomes with GdF > 3.0). In addition, periods estimated by Fourier transformation often lacked congruence with each other. Occasionally, robust periods measured for WW dinucleotides deviate from the genomic period, but this is expected given the Gaussian distribution of the periods of individual dinucleotides (Fig 4B and 4C).

### Selection for periodicity is conserved between closely-related genomes

**Fig 5:**
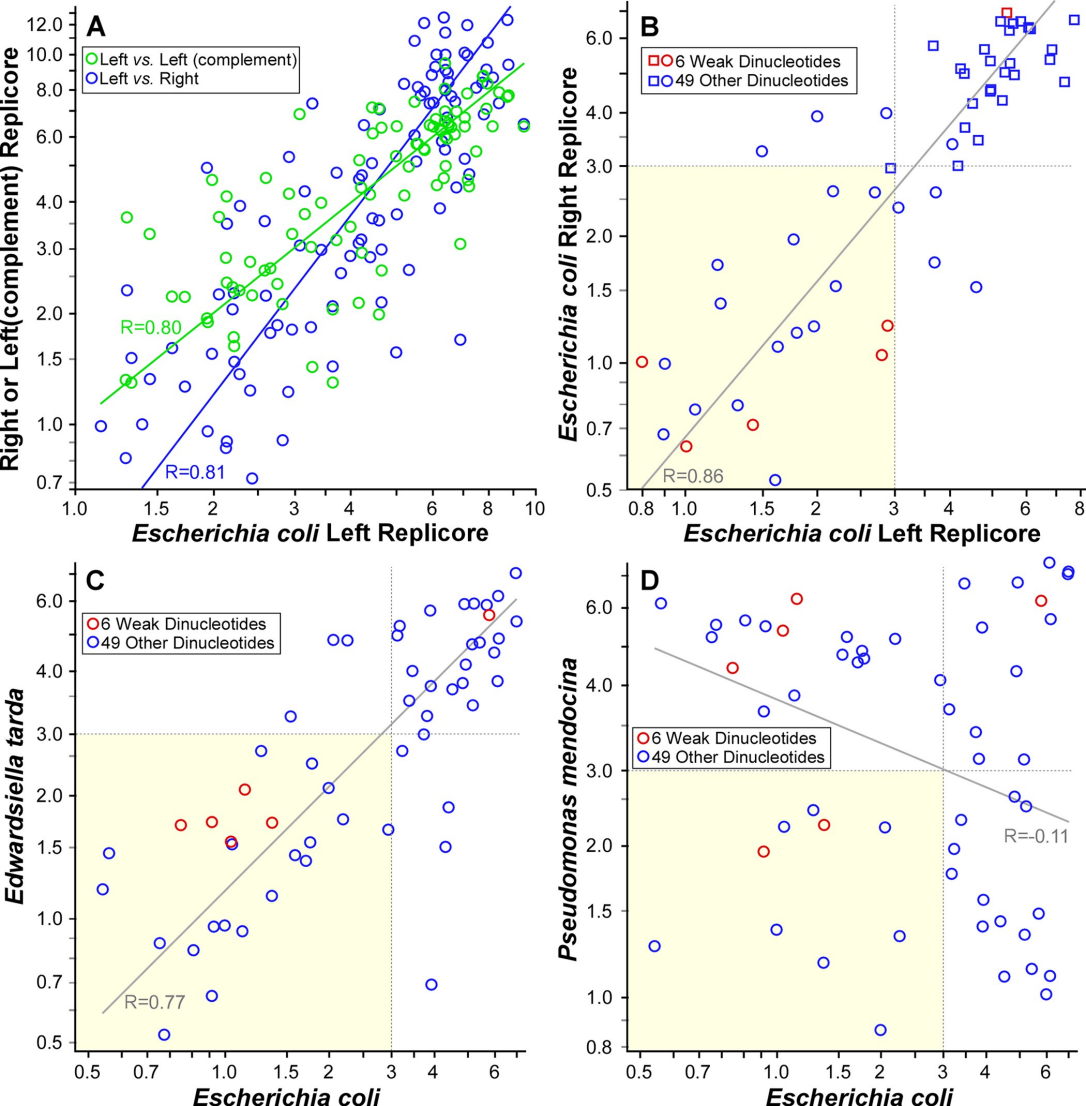
Periodic dinucleotides are conserved. (A) Goodnesses of fit for 100 dinucleotides on the right replicore (blue) and complement of the left replicore (green) in the *E*. *coli* K12 DH10b genomes plotted against GdF values for the left replicore. (B) Goodness of fit for 55 palindromic or complementary pairs of dinucleotides on the right replicore of the *E*. *coli* K12 DH10b genome plotted against values for the left replicore. Bend-inducing dinucleotides are indicated in red. The yellow shaded box indicates dinucleotides that are significantly periodic in both replicores. (C) Goodness of fit of 55 palindromic and complementary pairs of dinucleotides in *E*. *tarda* EIB202 as a function of goodness of fit in *E*. *coli*. (D) Goodness of fit of 55 palindromic and complementary pairs of dinucleotides in *P*. *mendocina* ymp as a function of goodness of fit in *E*. *coli*. In all cases, Deming regressions are plotted as solid lines.

The 100 dinucleotides tested include 10 palindromes (*e*.*g*., GC) and 45 complementary pairs (*e*.*g*., GA and TC). To verify that complementary pairs of dinucleotides are either both periodic or both non-periodic, we assessed periodicity of each of the 100 dinucleotides on the *E*. *coli* right replicore, left replicore, and complement of the left replicore (Fig 5A). As expected, the goodnesses of fit for individual dinucleotides were correlated between the left and right replicores (R = 0.81), meaning that dinucleotides that were periodic in one replicore were also periodic in the other replicore. Notably, the GdFs were also correlated between the left replicore and its complement to a comparable degree (R = 0.80). Because a dinucleotide and its complement have the same degree of periodicity, we henceforth combined spacings for the 45 pairs of complementary dinucleotides, yielding a total of 55 palindromic and complementary pairs of dinucleotides (Fig 5B).

Of these 55 dinucleotides in *E*. *coli*, 27 are significantly periodic when analyzing the entire genome (see Fig 5C and 5D), including 8 palindromes and 19 complementary pairs, 20 of which show significant periodicity even when using the reduced data sets of a single chromosome arm (Fig 5B). As expected, the significance of periodicity (the GdF of the curve fit) is shared between the left and right replicores (R = 0.86; Fig 5B). Only 5 of the 27 significantly periodic dinucleotides play a role in DNA bending, and 1 dinucleotide participating in DNA bending (TA) is not periodic in *E*. *coli* (see red points in Fig 5B). While it was speculated that WW dinucleotides were periodic by virtue of their impact on DNA bending in the absence of interacting partners, it is not clear what function is served by non-WW dinucleotides whose distributions are selected for periodicity. If they interact with a partner (*e*.*g*., a protein or small RNA), that partner may differ between organisms so that the suite of dinucleotides showing significant periodicity may become less similar as organisms become more distantly related.

To determine how the suites of periodic dinucleotides differ between organisms, we measured the significance of periodicity in each of the 55 dinucleotides in *Edwardsiella tarda* (like *E*. *coli*, a member of the Enterobacteriaceae) and *Pseudomonas mendocina* (in the Pseudomonadaceae). Similar suites of dinucleotides are significantly periodic in the two enteric bacteria, *E*. *coli* and *E*. *tarda* (R = 0.77; Fig 5C); this similarity does not reflect the lack of opportunity for mutation to act as only 2333 genes are homologous between these taxa (62% of *E*. *tarda* genes and 55% of *E*. *coli* genes), and synonymous sites of shared genes have each experienced more than one substitution (average K_S_ of shared genes is 1.445). A total of 23 dinucleotides (74% of significantly periodic dinucleotides) are periodic in both taxa, 24 dinucleotides are periodic in neither taxon, and only 8 are periodic in only one of the two taxa (26% of significantly periodic dinucleotides). This congruence of the suites of periodic dinucleotides between the species is only modestly lower than that between the two *E*. *coli* replicores (R = 0.77 vs. R = 0.86).

In contrast, *E*. *coli* and *P*. *mendocina* are in different families and have quite different suites of periodic dinucleotides (R = -0.11, Fig 5D). Here, only 10 dinucleotides are periodic in both taxa (24% of significantly periodic dinucleotides) and 14 dinucleotides are not periodic in either taxon, yet 31 dinucleotides– 76% of significantly periodic dinucleotides–are periodic in only one of the two taxa. Therefore, the suite of dinucleotides whose distributions are under selection for periodicity in *E*. *coli* are not representative of which dinucleotides are under selection for periodicity in other organisms.

### Similarity in periodicity correlates with phylogenetic distance

As shown in Fig 5, bacterial species in the same family share suites of significantly periodic dinucleotides, whereas those in different families do not. This suggests that differences in selection for periodicity in dinucleotide abundance will increase with phylogenetic distance between the organisms. That is, both the difference in the genomic period, and differences in the suites of periodic dinucleotides, are expected to increase as organisms become more distantly related.

**Fig 6:**
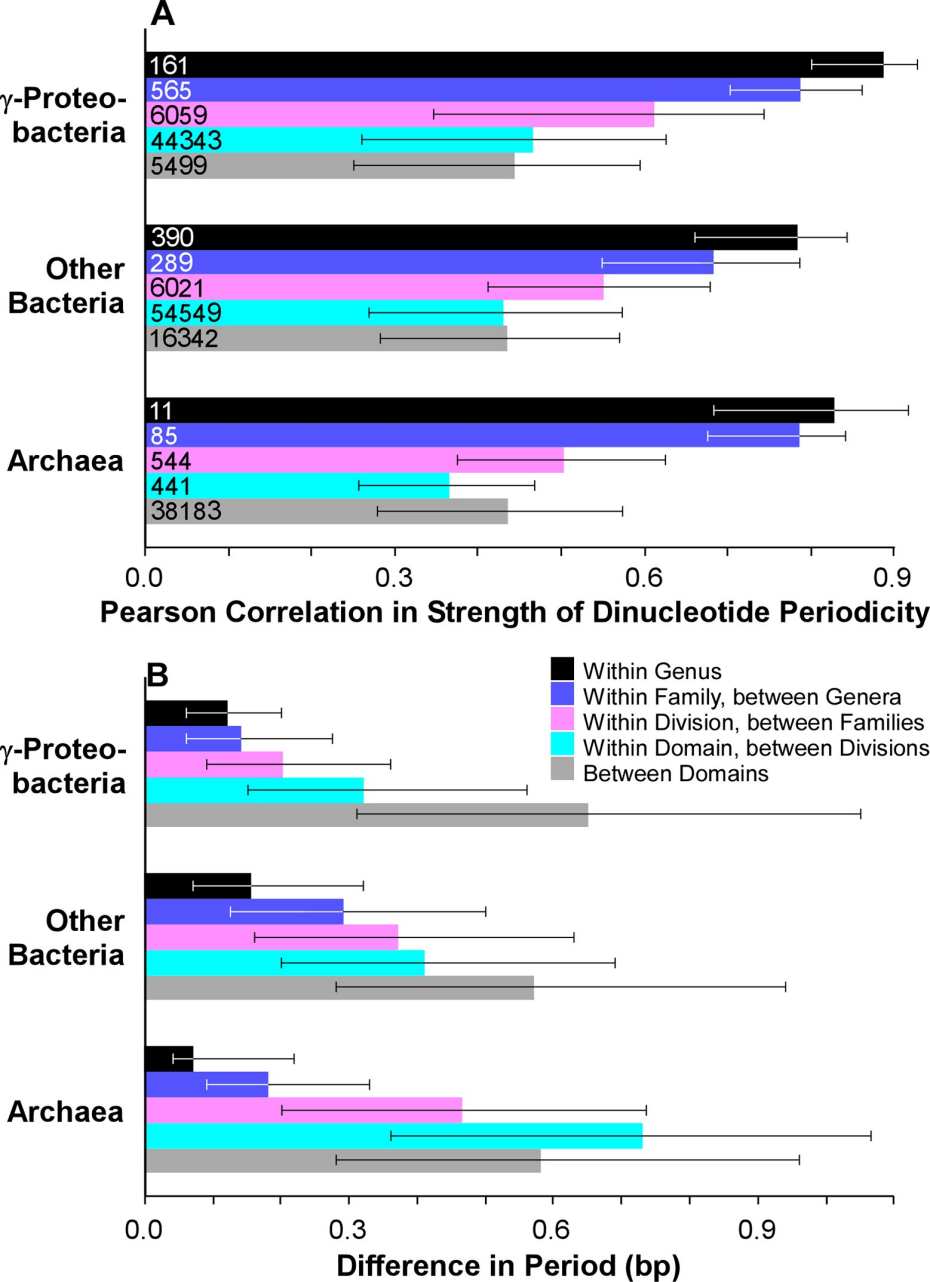
More closely related organisms have more similar genomic periodicity. (A) Median pairwise Pearson correlation between genomes at various levels of relatedness. (B) Median pairwise difference in period between genomes at various levels of relatedness. Bars extend to the medians of values above and below the overall sample median. Archaea and γ-proteobacteria were separated from all other Bacteria due to under- and over-sampling, respectively.

To test this hypothesis, we performed pairwise comparisons between 543 prokaryotic genomes with significant genomic periods (*i*.*e*., GdF < 3.0); all genomes showed less than 97% 16S rRNA similarity with other genomes in this data set, ensuring independence of measurements. As expected, different species in the same genus showed the greatest similarity in their suites of periodic dinucleotides (median R = 0.81, Fig 6A). Species in different genera of the same family were more different (median R = 0.76, Fig 6A), while species in the same division yet different families were more different still (median R = 0.56, Fig 6A). This trend was true for Archaea and Bacteria (Fig 6A). Differences in the genomic period also increased with phylogenetic distance for both Bacteria and Archaea (Fig 6B). The lower similarity in suites of periodic dinucleotides between more distantly-related taxa were not a result of increasingly different GC-contents of those genomes; when the data sets were normalized to have the same distributions of differences in GC-content, the same results were obtained (S5 Table). Therefore, we conclude that more closely-related organisms are more likely to share similarities in selection for periodicity, perhaps originating from shared protein or small-RNA interacting partners which utilize that information.

If suites of periodic dinucleotides for distantly-related organisms were selected at random from all 55 palindromic or complement-paired dinucleotides, then correlation between their strength of periodicity would be, on average, zero. Yet strikingly, while similarity in these suites of periodic dinucleotides decreases with phylogenetic distance, the median similarity remains significantly greater than zero even when comparing species between different domains (Fig 6A). This suggests that while selection favors periodicity in abundance between different suites of dinucleotides in different taxa, those suites consistently either include or exclude certain dinucleotides, thus leading to more similarity in these sets than expected at random.

### A shared genomic architecture selects for periodic distributions of dinucleotides in all organisms

**Fig 7:**
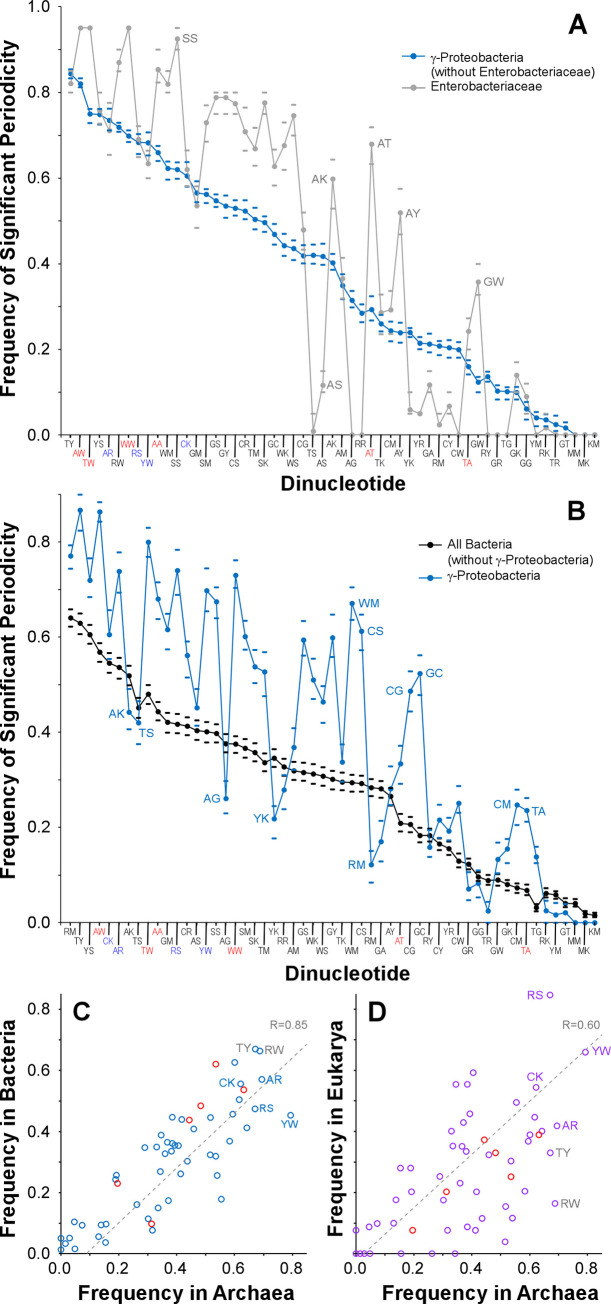
Periodicity is an ancient architecture predating the division of all known life. Mean percentage of genomes in which each dinucleotide is periodic. Bend-inducing dinucleotides are labeled in red. (A) Enterobacteriaceae and non-Enteric γ-proteobacteria are compared; to avoid oversampling, each genus was sampled once for each of 1500 iterations. Error bars represent one standard deviation (B) γ-proteobacteria and non- γ-proteobacteria Bacteria are compared; each family was sampled once for each of 1500 iterations. Error bars represent one standard deviation above or below the mean. (C) Correlation between percentage of Bacterial and Archaeal genomes in which each dinucleotide is periodic; each family was sampled once for each of 1500 iterations. The dotted grey line represents a Deming regression between the two Domains. (D) Correlation between percentage of Eukaryotic and Archaeal genomes in which each dinucleotide is periodic; each family was sampled once for each of 1500 iterations. The dotted grey line represents a Deming regression between the two Domains.

To determine if particular dinucleotides are consistently selected, or not selected, for periodic distributions, we determined which of the 55 dinucleotides were significantly periodic (GdF<3.0) in 68 genera of γ-proteobacteria (Fig 7A). Some dinucleotides were found to be periodic in many genera of γ-Proteobacteria (blue line in Fig 7A), with 6 dinucleotides being significantly periodic in more than 70% of genera; only two of these six dinucleotides are associated with DNA bending (red dinucleotides in Fig 7). In addition, some dinucleotides were rarely periodic in genera of γ-Proteobacteria, including 9 dinucleotides which are significantly periodic in less than 10% of genera. While the most commonly, and least commonly, periodic dinucleotides were shared between Enterobacteriaceae and all other γ-proteobacteria, notable departures from the Division average were observed in several of the dinucleotides of intermediate frequency within this family (compare grey and blue curves in Fig 7A). This is expected, as Fig 6 shows greater similarity in the suites of periodic dinucleotides for narrower taxonomic groups. We repeated this analysis for families within the domain Bacteria (Fig 7B). Here, 7 dinucleotides were significantly periodic in a representative from more than half of all families, and 13 dinucleotides were periodic in less than 10% of families. (Fig 7B). As expected from Fig 6, particular dinucleotides were more commonly periodic in individual divisions (*e*.*g*., the γ-Proteobacteria, Fig 7B).

Taken together, these data support the hypothesis that particular dinucleotides are consistently selected, or not selected, for periodic distributions, and those sets may be shared among species within the same genus, family, division, or domain. For each of the 55 palindromic or complementary pairs of dinucleotides, we assessed the frequency that the dinucleotide was periodic in a representative of each family of Bacteria, Archaea or Eukarya. Those frequencies were strongly correlated between Bacteria and Archaea (Fig 7C) and between Eukarya and Archaea (Fig 7D), with some dinucleotides being commonly significantly periodic, and some being rarely periodic, across all domains of Life. Notably, these commonly periodic dinucleotides are not those which are involved in DNA bending. Therefore, we propose that genomic periodicity is used for an important, fundamental mechanism that predates the diversification of all known life.

### Periodicity is characteristic of the chromosome

Previous work suggested that regions of the chromosome showing periodic dinucleotide distributions may be confined to relatively few loci, with the majority of the chromosome not exhibiting the genomic period [22], which was congruent with the limited number of sites known to show strong DNA bending [29]. However, our data suggest that dinucleotides involved in DNA bending are not those that are most frequently periodic in Bacterial, Archaeal, or Eukaryotic genomes (Fig 7), which casts doubt on the suggestion that few loci in a genome show periodicity.

**Fig 8:**
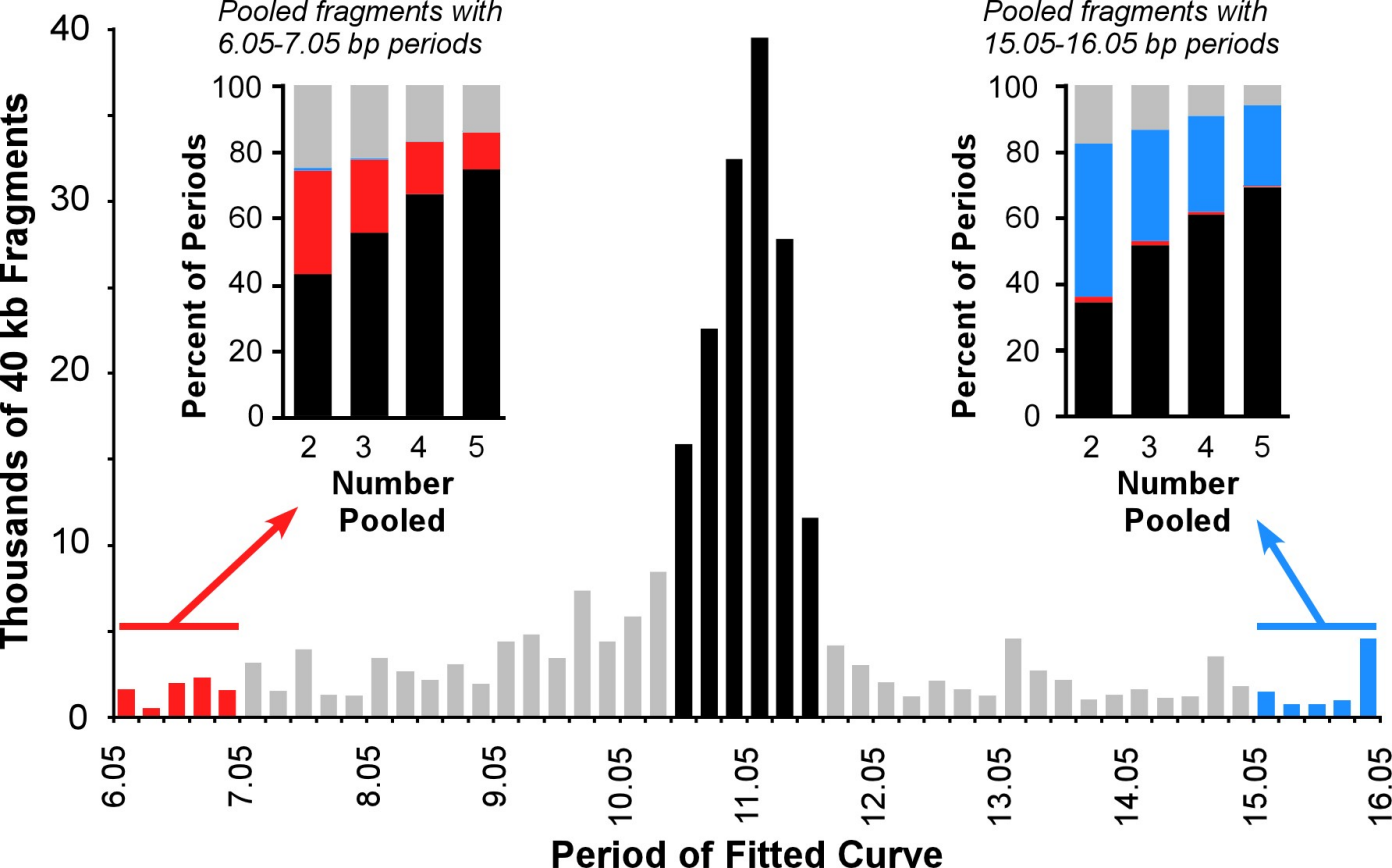
Periodicity is characteristic of the chromosome. Distribution of periods for best-fit curves of the autocorrelations of dinucleotide spacings of 270,000 random 40 kb fragments of the *E*. *coli* K12 DH10B genome; the curve-fitting algorithm explored periods within 5 bp of the genomic period (6.05 to 16.05 bp). Fragments within 0.6 bp of the genomic period (11.05 bp) are shaded black, fragments in the lowest (6.05–7.05 bp) and highest (15.05–16.05) ranges of periods explored are shown in red and blue, respectively. The insets show percentages of estimated periods for sequences generated by pooling 2, 3, 4, or 5 fragments initially showing periods within the lowest (left inset) or highest (right inset) range of explored periods. Bars in the insets are color-coded to the ranges denoted in the primary figure.

To determine whether selection for periodicity is limited to few loci, we selected 270,000 randomly positioned 40 kb segments within the *E*. *coli* K12 DH10B genome and analyzed them for periodicity (Fig 8). To decrease noise, we collated only the spacings of the palindromic and complementary pairs of dinucleotides with strong periodicity (GdF < 1.5, amplitude > 0.2). The majority of fragments (55%) exhibited inferred periods within 0.6 bp of the genomic period, 11.05 bp (black bars in Fig 8), with the remaining fragments showing periods within the range explored (6.05–16.05 bp). The minority of 40 kb fragments which show aberrant periods either (a) lack regions under selection for the genomic period and report a stochastic, non-robust period, (b) have a true robust period that differs from the genomic period, or (c) have regions under selection for the genomic period, but this information is too subtle to be detected and the genomic period cannot be inferred.

To discriminate between these hypotheses, we catenated two to five non-overlapping fragments which initially showed extreme periods of 6.05–7.05 bp (Fig 8, left inset) or 15.05–16.05 bp (Fig 8, right inset). If these fragments lack selection for periodicity, we expect catenated samples to have a variety of periods because the period estimated for each fragment was not significant. If these fragments bore a true period that is different from the genomic period, we expect that catenated samples would reinforce these non-genomic periods. However, we see that increasing fractions of catenated samples show the genomic period (11.05 +/- 0.6 bp) as more non-overlapping fragments are catenated (left and right insets in Fig 8); this was true for both regions analyzed. Therefore, we conclude that chromosomal regions that initially fail to show the genomic period have experienced selection for periodicity, but the strength of signal is too low to be detected by these methods.

Previous analysis of strength of periodicity did not yield the same conclusions; Mrázek [22] examined 10 kb fragments and found that only ~19% of the fragments lay within 0.6 bp of the genomic period (between 10.4 and 11.6 bp; S4A Fig). However, he analyzed a different strain of *E*. *coli*, considered a different range of periods (5 bp to 20 bp), examined only motifs associated with DNA bending, and determined the period using the power spectrum of a Fourier transformation. More importantly, he did not consider whether fragments which did not yield the genomic period simply had information below the threshold of detection. Therefore, we repeated the analysis shown in Fig 8 using the same genome as analyzed by Mrázek [22], but using our methods as applied to WW dinucleotides. We found a significantly larger fraction of 10 kb fragments (~30%) lay within 0.6 bp of the genomic period (S4B Fig). More importantly, fragments which initially showed aberrant periods (5–6 bp, or 17–18 bp, different ranges than analyzed in Fig 8) did show the genomic period when analyzed in aggregate (insets in S4B Fig), just as seen with 40 kb fragments in Fig 8. Therefore, we conclude that regions under selection for periodicity are distributed throughout the genome, even within fragments where the signal is too weak to be detected, and not concentrated in small sections of the chromosome.

## Discussion

### Potential roles for genomic periodicity

The finding that periodicity in dinucleotide motifs increases with the strength of codon selection demonstrates that these patterns are not simply the result of mutational biases; therefore, periodicity must serve some function. That function is likely ubiquitous and ancient because a common core of dinucleotide motifs is periodic across Bacteria, Archaea and Eukarya (Fig 7). This ubiquity suggests a fundamental biological function that has been conserved since the divergence of all known life. However, that role is unclear.

When only those motifs that imparted DNA bending were thought to be periodic, models for their function were centered on a possible role in DNA compaction. In *E*. *coli*, DNA compaction proteins IHF and HU preferentially bind AT-rich regions [30], and H-NS preferentially binds curved DNA [31]. However, the majority of periodic motifs in *E*. *coli* are neither AT-rich nor involved in DNA bending (Fig 4). The size of bend-prone A-rich regions periodically interposed with non-bend-prone regions was measured in *E*. *coli* to be 100–130 bp [22, 29]. Yet, local regions of periodicity in *E*. *coli* extend for at least a kilobase (S5 Fig). Both observations are inconsistent with a role for this periodicity in DNA compaction.

Reinforcing this point, some large, lytic phages–such as *Erwinia* phages Ea35-70 and vB_EamM_MadMel [32]–show periodicity even after limiting the analysis to protein-coding genes shared among groups of related bacteriophages and encoding proteins <70% identical to any known bacterial protein (S6 Fig). Being lytic phages, the genomes do not integrate into a bacterial chromosome nor do they employ HU or H-NS during DNA packaging into a capsid. Periodicity in lytic phages also suggests that the selection for periodicity does not arise from a role in DNA compaction or another cellular function such as chromosome segregation which would not operate on the phage genome. Because periodic motifs are distributed throughout genomes (Fig 8), this function must not be localized to a defined region, such as sequestration of replication origin. A binding partner is likely involved due to variability in which motifs are periodic; this variability belies a strict biophysical role for the periodic motifs in the absence of a partner, which would not vary between organisms (Fig 5D, Fig 6). Such functions could include sites for interaction with helicases, motor proteins, or other processive moieties such as those acting in in DNA translocation, DNA replication, or DNA repair.

### Impacts on genome evolution

Periodicity in dinucleotide motifs is evident if one examines only the sequences of protein-coding genes, ignoring any intergenic DNA (S7 Fig). Therefore, selection for periodicity has the potential to impact gene evolution as the same bases must convey multiple types of information, both residue and codon choice as well as periodic motifs. Thus, the rate of divergence in different genes may vary not only with selection for protein function and codon choice, but also with any variation in selection for periodicity within a genome (*e*.*g*., one interpretation of Fig 8). In addition, small insertions and deletions–either within or between genes–would be under compensatory selection to restore periodicity disrupted by a proximal insertion or deletion. That is, a small insertion or deletion may rise to high frequency in a population not due to its neutral or beneficial impact on the encoded protein, but because it restored periodicity that was disrupted by a proximal insertion or deletion. In this way, the existence and lengths of proximal insertions and deletions may not be independent, or be only constrained by the function of the local protein sequence. Testing these hypotheses is beyond the scope of this paper, but raises intriguing possibilities for the influence of genomic periodicity on gene evolution.

### Potential role in restriction of LGT

Regardless of function, periodicity is under selection across all domains of life. Yet the motifs under selection, and their periods, vary between genomes, with more closely-related genomes showing more similarity (Fig 6). Therefore, we would expect lateral gene transfer between distantly-related genomes to be disfavored as the incoming DNA would not embed the periodic signature of the recipient genome. As a result, recently acquired genomic islands should be more similar in periodicity to their recipient genomes than one would expect at random. This restriction on LGT from more distantly-related taxa has been shown to be mediated by other genomic architectures [33], where recently-acquired DNA fragments were more similar to their recipient genomes than expected at random. Hence, selection for periodic motifs has the potential to shape the flow of genes between taxa, thus shaping the genotypic and phenotypic characteristics of higher taxonomic groups [33].

### Variation of periodicity within a genome

The strength of periodicity varies within a genome (Fig 8). In *E*. *coli*, 65% of 40 kb fragments showed a period within 1 bp of the genomic period (Fig 8); those fragments were distributed throughout the genome (S8 Fig). Unexpectedly, fragments overlapping by more than 90% of their length may have dramatically different strengths of periodicity. Variation in strength of periodicity could be due to (a) small insertions or deletions that disrupt the spacings of otherwise periodically distributed motifs, (b) acquisition of foreign genes with slightly discordant periodicity, or (c) selection for other information that overwrites periodicity. Alternatively, the strength of selection for periodicity may vary within a genome at either long (kilobases) or short (dozens of bases) spatial scales. Discriminating among these alternatives may shed light on the function of these conserved, periodically-distributed motifs.

### Summary

Periodicity in dinucleotide motifs that impart DNA bending has been observed for decades [19, 22, 28, 29, 34–37]. However, contrary to being a relatively uniform pattern varying only in period by organismal growth temperature, we find that different motifs are periodic in different organisms, and their variability both in composition and in period bespeaks interaction with an ancient binding partner whose function has remained intact throughout the diversification of all known life. This variability has the potential to shape both gene and genome evolution in previously unappreciated ways.

## Materials and methods

### Genomes

All genome sequences were downloaded from NCBI; all analyses used the largest replicon from any one organism and used the gene annotations provided by the authors. Growth temperatures of organisms were kindly provided by Tatiparthi Balakrishna Reddy (DOE Joint Genome Institute) or obtained from the BacMap (http://bacmap.wishartlab.com/) and MicrobeWiki (https://microbewiki.kenyon.edu/index.php/MicrobeWiki) databases.

### Assessment of periodicity of nucleotide motifs

Within each sequence, we identified the locations of each instance of 100 dinucleotides (all dinucleotides composed of IUPAC bases G, A, T, C, R, Y, W, S, M, or K; complementary bases were assigned as C, T, A, G, Y, R, W, S, K and M, respectively). We collated the spacings between instances of each particular dinucleotide up to 202 bp apart. For analysis of individual replicores, origins and termini (*dif* site) were determined by homology to previously published locations [38–41]. To eliminate the three-base periodicity imparted by the genetic code, we averaged the spacings over three bases; thus, a 200 bp reported spacing is an average of those at 200, 201 and 202 bp.

To reduce noise, we calculated a continuous autocorrelation of the abundances of spacings between 6 bp (to reduce the impact of homopolymeric repeats) and 100 bp. A periodic distribution of motif spacings would lead to positive correlations of the abundance of spacings at integral multiples of the underlying period, and negative correlations between those maxima. However, small insertions and deletions would eliminate significant autocorrelations with increasing distance between motifs. Therefore, we fit a damped sine curve to the autocorrelation data, ignoring the first 9 intervals to again minimize the influence of nucleotide repeats. All analyses used the DNA Master software package, available at http://cobamide2.bio.pitt.edu.

### Fitting curves to autocorrelation data

We fit a damped sine curve to the autocorrelation data as:

$$Y=e^{-\frac{Ln(2)x}{HL}}A\;sin\left(\frac{2\pi x}{\lambda }+\frac{\pi }{2}\right)$$ Eq 1

where Y is the predicted autocorrelation value, x is the distance between spacings, λ is the period, A is the amplitude, HL is the half-life of the exponential decay, and where the phase was set as π/2. Best fits were obtained by exploring a range of values for A, HL and λ, then refining the estimates. The periods were typically explored in the range of 6 to 16 bp initially in 0.25 bp intervals, then in 0.01 bp intervals within 0.8 bp of the initial period estimate. The amplitude was explored from 0 to 120% of the maximum observed autocorrelation value in 0.05 increments, then refined in 0.02 increments within 20% of the initial amplitude estimate. The half-life was explored between 5 and 60 base pairs in 5 bp increments, then refined in 2% intervals over half-lives within 20% of the original estimate.

The best fit to the data was obtained by minimizing the difference (measured as a χ^2^) between the autocorrelation data and the predicted curve. The goodness of fit was calculated as a normalized χ^2^ as shown in Eq 2:

$$Goodness\;of\;fit=\frac{\chi^{2}}{\sigma }$$ Eq 2

where σ is the standard deviation of the genuine data. The strength of periodicity was calculated as the log transformed quotient of the Goodness of Fit (GdF) and the Amplitude–Ln(GdF/A)–where smaller values indicate better fits with greater autocorrelation values. For comparison of the strength of periodicity between genomes or different lengths, the same total numbers of dinucleotide spacings were used.

### Determining the variance of the period

The spacing data were partitioned into 1000 subsets, such that each subset represents 0.1% of the spacings distributed throughout the region analyzed. As instances of each dinucleotide were compared to identify those with spacings up to 202 bp, each spacing within this range was placed in one of 1000 subsets, with a different subset chosen for each subsequent spacing identified. The genuine period was calculated by collating all 1000 subsets, performing an autocorrelation, and fitting a damped sine curve to those data. In contrast, a bootstrapped data set can be created by collating spacing data from 1000 subsets chosen at random with replacement; a curve can be fit to the resulting autocorrelation. Variance in the genomic period was calculated by repeating the bootstrapping procedure 1000 times, and the variance of that distribution of periods was used to represent the variance of the genuine period [42]. That variance did not appreciably change for division of the data into 50 or more subsets, so a value of 1000 subsets was chosen. The variance of the period of individual dinucleotides was calculated the same way; significance of the difference of the periods of individual dinucleotides from the genomic period was tested using a Student *t*-test.

### Measurement of divergence

Orthologous genes were identified as reciprocal best BLAST hits between cognate proteins with 80% minimal amino acid similarity with the next best match having no more than 40% amino acid similarity. For orthologous genes, divergence at synonymous and nonsynonymous sites was calculated using the method of Li [43]. Genomic average values for K_A_ and K_S_ were calculated as the arithmetic mean of individual divergence values weighted by the length of the aligned sequences.

### Measurement of codon selection

Strength of codon selection within a genome was measured using both ACE- χ^2^ and ENC’ [25, 26]. For each genome, codon frequencies under strong codon selection were inferred from the subset present of 40 genes whose products are involved in translation [27]; codon frequencies in the absence of strong codon selection were inferred from core genomes having eliminated the 20% of genes with the most atypical codon usage or codon-position-specific dinucleotide frequencies [26]. To compare the strength of codon selection between genomes, ACE- χ^2^ and ENC’ were calculated using the subset of genes shared among genomes being compared.

### Examining dinucleotides

When determining how frequently a dinucleotide is periodic, oversampling of some taxa can lead to biased measures. To assess how frequently a dinucleotide was periodic within a Division, we chose a genome at random from each genus, selecting from a set of genomes with less than 97% 16S rRNA similarity. We repeated random selection of genomes from each genus for 1500 iterations and found the mean and variance of the frequencies each of the 55 dinucleotides were significantly periodic in exemplars of the genera. To assess how frequently a dinucleotide was periodic within a Domain, we repeated this procedure selecting a genome at random from each family.

### Data

Data have been deposited in the Dryad repository: http://dx.doi.org/10.5061/dryad.0p2ngf1zf [44].

## Supporting information

S1 Table Distribution of goodnesses of fit for damped sines fit to randomized autocorrelation data. (DOCX)

S2 Table Periods of *E*. *coli* dinucleotides do not differ significantly from the genomic period. (DOCX)

S3 Table Kolmogorov-Smirnov tests for normality of the distribution of individual dinucleotide periods. (DOCX)

S4 Table Comparison of genomic periods inferred by different methods. (XLSX)

S5 Table Comparison of periodicity between genomes of different degrees of relatedness, normalized by difference in GC content. (DOCX)

S1 Fig Variance in the genomic period is predicted by the goodness of fit. The goodness of fit to the curve fit to autocorrelation data was calculated for 764 genomes, none with 16S rRNA similarity greater than 97%. The variance in the genomic period was estimated by 1000 bootstrap resamples of the dinucleotide spacings.
(TIF)

S2 Fig Artificial genomes lack periodicity. Autocorrelations of the abundances of spacings from both the genuine genome (orange) and from an artificial genome with the same codon-position-specific dinucleotide, trinucleotide and tetranucleotide frequencies, genes length distribution, and strand bias (blue) are shown for (A) *E*. *coli*, (B) *A*. *fulgidus*, and (C) *H*. *influenzae*.
(TIF)

S3 Fig Periodicity is not imparted by protein motifs. Autocorrelations of dinucleotide spacings were calculated for 27 genomes using all spacings (set I), or calculated by omitting either spacings between pairs of dinucleotides at the first and second codon positions within the same genes (set II), or spacings between pairs of dinucleotides at the third and first codon positions within the same genes (set III). Damped sine curves were fit to all three data sets. (A) Differences in goodness of fit between sets I and II (blue) or I and III (red). (B) Differences in amplitude between sets I and II (blue) or I and III (red). (C) Differences in period between sets I and II (blue) or I and III (red). The mean (μ) and standard deviation (σ) of the distributions of differences for the 27 genomes are displayed.
(TIF)

S4 Fig Periodicity is characteristic of the chromosome. (A) Periods of sequential 10 kb fragments from the *E*. *coli* K12 MG1655 genome, stepping 5 kb, estimated from the power spectrum of Fourier transformation; data are replotted from Mrázek [22] Fig 2B, combining data from 0.1 bp intervals to 0.2 bp intervals. Fragments within 0.6 bp of the genomic period (11.05 bp) are shaded black. (B) Distribution of periods for best-fit curves of the autocorrelations of dinucleotide spacings of 270,000 random 10 kb fragments of the *E*. *coli* K12 MG1655 genome. Fragments within 0.6 bp of the genomic period (11.05 bp) are shaded black, fragments with periods estimated in the lower (5.0–6.0 bp) and higher (17.0–18.0 bp) ranges are shown in red and blue, respectively. The insets show percentages of estimated periods for sequences generated by pooling 2, 3, or 5 fragments initially showing periods within the lower (left inset) or higher (right inset) range of explored periods. Bars in the insets are color-coded to the ranges denoted in the primary figure.
(TIF)

S5 Fig Periodicity in the *E*. *coli* DH10B genome extends for at least 1000 bp. Autocorrelation of the abundances of spacings between cognate dinucleotides. Spacings were measured up to 1 kb apart; autocorrelations were calculated for differences in spacings up to 500 bp (see methods). Data are shown in blue and the best fit of a damped sine curve fit to these data is shown in red.
(TIF)

S6 Fig Periodicity in lytic phage genomes. Autocorrelation of the abundances of spacings between cognate dinucleotides calculated for protein-coding genes shared between *Erwinia* phages Ea35-70 and vB_EamM_MadMel, removing two genes with >70% identity of their inferred proteins with bacterial genes. Spacings were measured up to 200 bp apart; autocorrelations were calculated for differences in spacings up to 100 bp (see methods). Data are shown in blue and the best fit of a damped sine curve fit to these data is shown in red.
(TIF)

S7 Fig Sequences within open reading frames show dinucleotide periodicity. Autocorrelation of the abundances of spacings within *E*. *coli* open reading frames, with a damped sine curve fit to these data. Autocorrelation of the abundances of spacings within the entire *E*. *coli* genome (data from Fig 1) are included for comparison.
(TIF)

S8 Fig The strength of periodicity varies within a genome. Distribution along the *E*. *coli* chromosome of 40 kb segments whose periods were estimated to be within 0.0 to 0.5 bp of the genomic period (red) and within 0.5 to 1.0 bp of the genomic period (blue) plotted as its goodness of fit to a damped sine curve. Darker shading indicates a higher amplitude (stronger periodicity).
(TIF)
