# Supplementary material for: Selection for ancient periodic motifs that do not impart DNA bending
Source: PLoS Genet. 2020 Oct 6;16(10):e1009042. doi: 10.1371/journal.pgen.1009042 (PMC7537859; doi:10.1371/journal.pgen.1009042)
Supplement: S1 Table — (DOCX) [file pgen.1009042.s001.docx]

**S1 Table. Distribution of goodnesses of fit for damped sines fit to randomized autocorrelation data.**

| Goodness of Fit | *Escherichia coli* | *Mycoplasma genitalium* | *Buchnera aphidicola* | *Bacteroides xylanisolvens* | *Ehrlichia ruminantium* | *Methanococcus maripaludis* | *Geodermatophilus obscurus* | Total |
| --- | --- | --- | --- | --- | --- | --- | --- | --- |
| Number of Randomization Replicates | | | | | | | | |
| 2.5 | 1 | 0 | 0 | 0 | 0 | 0 | 0 | 1 |
| 3 | 5 | 4 | 3 | 4 | 7 | 5 | 0 | 28 |
| 3.5 | 32 | 31 | 25 | 34 | 34 | 44 | 37 | 237 |
| 4 | 268 | 241 | 249 | 276 | 254 | 223 | 299 | 1810 |
| 4.5 | 1221 | 1174 | 1165 | 1200 | 1235 | 1121 | 1201 | 8317 |
| 5 | 2870 | 2864 | 2876 | 2800 | 2804 | 2740 | 2813 | 19767 |
| 5.5 | 3117 | 3143 | 3132 | 3171 | 3163 | 3212 | 3149 | 22087 |
| 6 | 1760 | 1792 | 1807 | 1763 | 1777 | 1838 | 1756 | 12493 |
| 6.5 | 562 | 575 | 593 | 589 | 564 | 615 | 579 | 4077 |
| 7 | 126 | 141 | 131 | 130 | 132 | 168 | 136 | 964 |
| 7.5 | 32 | 29 | 17 | 23 | 28 | 27 | 24 | 180 |
| 8 | 5 | 4 | 2 | 10 | 1 | 5 | 5 | 32 |
| 8.5 | 1 | 2 | 0 | 0 | 1 | 2 | 1 | 7 |
| Count | 10000 | 10000 | 10000 | 10000 | 10000 | 10000 | 10000 | 70000 |
| Mean | 5.11 | 5.12 | 5.12 | 5.12 | 5.11 | 5.14 | 5.11 | 5.12 |
| Variance | 0.37 | 0.37 | 0.36 | 0.38 | 0.37 | 0.38 | 0.38 | 0.37 |
|  |  |  |  |  |  |  |  |  |
| Cumulative Distribution (Percent) | | | | | | | | |
| 2.5 | 0.01 | 0.00 | 0.00 | 0.00 | 0.00 | 0.00 | 0.00 | 0.00 |
| 3 | 0.06 | 0.04 | 0.03 | 0.04 | 0.07 | 0.05 | 0.00 | 0.04 |
| 3.5 | 0.4 | 0.4 | 0.3 | 0.4 | 0.4 | 0.5 | 0.4 | 0.4 |
| 4 | 3.1 | 2.8 | 2.8 | 3.1 | 3.0 | 2.7 | 3.4 | 3.0 |
| 4.5 | 15.3 | 14.5 | 14.4 | 15.1 | 15.3 | 13.9 | 15.4 | 14.8 |
| 5 | 44.0 | 43.1 | 43.2 | 43.1 | 43.3 | 41.3 | 43.5 | 43.1 |
| 5.5 | 75.1 | 74.6 | 74.5 | 74.9 | 75.0 | 73.5 | 75.0 | 74.6 |
| 6 | 92.7 | 92.5 | 92.6 | 92.5 | 92.7 | 91.8 | 92.6 | 92.5 |
| 6.5 | 98.4 | 98.2 | 98.5 | 98.4 | 98.4 | 98.0 | 98.3 | 98.3 |
| 7 | 99.6 | 99.7 | 99.8 | 99.7 | 99.7 | 99.7 | 99.7 | 99.7 |
| 7.5 | 99.9 | 99.9 | 100.0 | 99.9 | 100.0 | 99.9 | 99.9 | 99.9 |
| 8 | 100.0 | 100.0 | 100.0 | 100.0 | 100.0 | 100.0 | 100.0 | 100.0 |
| 8.5 | 100.0 | 100.0 | 100.0 | 100.0 | 100.0 | 100.0 | 100.0 | 100.0 |
