# Supplementary material for: Selection for ancient periodic motifs that do not impart DNA bending
Source: PLoS Genet. 2020 Oct 6;16(10):e1009042. doi: 10.1371/journal.pgen.1009042 (PMC7537859; doi:10.1371/journal.pgen.1009042)
Supplement: S2 Table — (DOCX) [file pgen.1009042.s002.docx]

**S2 Table**. Testing difference between periods of individual dinucleotide and the genomic period.

|  | Genuine Genome | | |  | Bootstrap | |  | Comparison to Genomic Period | |
| --- | --- | --- | --- | --- | --- | --- | --- | --- | --- |
| DN | Period | Amplitude | Goodness of Fit |  | Mean | Standard Deviation |  | t | P |
| AR | 10.89 | 0.386 | 1.494 |  | 10.89 | 0.061 |  | -3.038 | 0.003 |
| TW | 11.03 | 0.437 | 0.634 |  | 11.03 | 0.021 |  | -1.841 | 0.069 |
| CK | 11.16 | 0.430 | 1.165 |  | 11.15 | 0.047 |  | 1.619 | 0.109 |
| SK | 11.14 | 0.502 | 0.612 |  | 11.14 | 0.041 |  | 1.607 | 0.111 |
| WK | 10.96 | 0.443 | 1.315 |  | 10.96 | 0.074 |  | -1.574 | 0.119 |
| YS | 10.93 | 0.225 | 1.162 |  | 10.93 | 0.094 |  | -1.495 | 0.138 |
| SM | 10.99 | 0.391 | 1.494 |  | 10.97 | 0.067 |  | -1.486 | 0.140 |
| WW | 11.04 | 0.352 | 0.592 |  | 11.04 | 0.018 |  | -1.438 | 0.154 |
| AW | 11.05 | 0.435 | 0.414 |  | 11.05 | 0.020 |  | -1.224 | 0.224 |
| AA | 11.03 | 0.557 | 0.935 |  | 11.03 | 0.037 |  | -1.197 | 0.234 |
| GM | 10.88 | 0.304 | 2.184 |  | 10.84 | 0.222 |  | -1.054 | 0.295 |
| RS | 11.11 | 0.601 | 0.751 |  | 11.11 | 0.039 |  | 0.942 | 0.349 |
| GS | 11.05 | 0.405 | 1.285 |  | 11.04 | 0.034 |  | -0.903 | 0.369 |
| SS | 11.05 | 0.321 | 0.838 |  | 11.05 | 0.025 |  | -0.840 | 0.403 |
| TY | 11.11 | 0.515 | 1.324 |  | 11.11 | 0.052 |  | 0.721 | 0.473 |
| RW | 11.09 | 0.470 | 0.925 |  | 11.09 | 0.032 |  | 0.529 | 0.598 |
| AK | 11.03 | 0.307 | 1.882 |  | 11.01 | 0.119 |  | -0.520 | 0.604 |
| AT | 11.11 | 0.280 | 1.322 |  | 11.10 | 0.058 |  | 0.410 | 0.682 |
| GY | 11.10 | 0.418 | 1.405 |  | 11.10 | 0.066 |  | 0.335 | 0.738 |
| WM | 11.09 | 0.433 | 1.117 |  | 11.09 | 0.035 |  | 0.311 | 0.756 |
| CS | 11.09 | 0.295 | 1.044 |  | 11.09 | 0.048 |  | 0.304 | 0.762 |
| YW | 11.06 | 0.649 | 0.890 |  | 11.06 | 0.073 |  | -0.248 | 0.805 |
| GC | 11.08 | 0.297 | 1.584 |  | 11.06 | 0.088 |  | -0.129 | 0.897 |
