# Supplementary material for: Selection for ancient periodic motifs that do not impart DNA bending
Source: PLoS Genet. 2020 Oct 6;16(10):e1009042. doi: 10.1371/journal.pgen.1009042 (PMC7537859; doi:10.1371/journal.pgen.1009042)
Supplement: S3 Table — (DOCX) [file pgen.1009042.s003.docx]

**S3 Table**. Kolmogorov-Smirnov tests for normality of the distribution of individual dinucleotide periods.

|  |  |  | **Kolmogorov-Smirnov Test** | | | | |
| --- | --- | --- | --- | --- | --- | --- | --- |
|  |  |  | **Periodic** |  | **Limit** | **Limit** | **Limit** |
| **Organism** | **Division** | **Family** | **DNs** | **Dn** | **α=0.1** | **α=0.05** | **α=0.01** |
| Acetohalobium arabaticum DSM 5501 | Firmicutes | Halobacteroidaceae | 32 | 0.172 | 0.211 | 0.234 | 0.281 |
| Acholeplasma laidlawii PG-8A | Tenericutes | Acholeplasmataceae | 23 | 0.150 | 0.248 | 0.275 | 0.330 |
| Acidiphilium cryptum JF-5 | Alphaproteobacteria | Acetobacteraceae | 19 | 0.099 | 0.271 | 0.301 | 0.361 |
| Acidithiobacillus ferrooxidans ATCC 23270 | Gammaproteobacteria | Acidithiobacillaceae | 12 | 0.259 | 0.338 | 0.375 | 0.449 |
| Acinetobacter sp. DR1 | Gammaproteobacteria | Moraxellaceae | 29 | 0.088 | 0.221 | 0.246 | 0.295 |
| Actinobacillus pleuropneumoniae L20 | Gammaproteobacteria | Pasteurellaceae | 33 | 0.059 | 0.208 | 0.231 | 0.277 |
| Aerococcus urinae ACS-120-V-Col10a | Firmicutes | Aerococcaceae | 25 | 0.058 | 0.238 | 0.264 | 0.317 |
| Aeromonas hydrophila subsp. hydrophila ATCC 7966 | Gammaproteobacteria | Aeromonadaceae | 34 | 0.089 | 0.205 | 0.228 | 0.273 |
| Aggregatibacter actinomycetemcomitans D11S-1 | Gammaproteobacteria | Pasteurellaceae | 31 | 0.140 | 0.214 | 0.238 | 0.285 |
| Agrobacterium vitis S4 | Alphaproteobacteria | Rhizobiaceae | 34 | 0.159 | 0.205 | 0.228 | 0.273 |
| Alcanivorax borkumensis SK2 | Gammaproteobacteria | Alcanivoracaceae | 31 | 0.066 | 0.214 | 0.238 | 0.285 |
| Aliivibrio salmonicida LFI1238 | Gammaproteobacteria | Vibrionaceae | 19 | 0.109 | 0.271 | 0.301 | 0.361 |
| Allochromatium vinosum DSM 180 | Gammaproteobacteria | Chromatiaceae | 20 | 0.160 | 0.265 | 0.294 | 0.352 |
| Alteromonas macleodii 'Deep ecotype' | Gammaproteobacteria | Alteromonadaceae | 30 | 0.161 | 0.218 | 0.242 | 0.290 |
| Ammonifex degensii KC4 | Firmicutes | Thermoanaerobacteraceae | 15 | 0.118 | 0.304 | 0.338 | 0.404 |
| Amycolatopsis mediterranei U32 | Actinobacteria | Pseudonocardiaceae | 31 | 0.097 | 0.214 | 0.238 | 0.285 |
| Anabaena variabilis ATCC 29413 | Cyanobacteria | Nostocaceae | 13 | 0.098 | 0.325 | 0.361 | 0.432 |
| Anaerocellum thermophilum DSM 6725 | Firmicutes | Unknown | 18 | 0.108 | 0.279 | 0.309 | 0.371 |
| Anaerococcus prevotii DSM 20548 | Firmicutes | Unknown | 34 | 0.079 | 0.205 | 0.228 | 0.273 |
| Anaplasma centrale str. Israel | Alphaproteobacteria | Anaplasmataceae | 23 | 0.118 | 0.248 | 0.275 | 0.330 |
| Aquifex aeolicus VF5 | Aquificae | Aquificaceae | 25 | 0.088 | 0.238 | 0.264 | 0.317 |
| Arcanobacterium haemolyticum DSM 20595 | Actinobacteria | Actinomycetaceae | 27 | 0.104 | 0.229 | 0.255 | 0.305 |
| Aromatoleum aromaticum EbN1 | Betaproteobacteria | Rhodocyclaceae | 23 | 0.211 | 0.248 | 0.275 | 0.330 |
| Atopobium parvulum DSM 20469 | Actinobacteria | Coriobacteriaceae | 26 | 0.192 | 0.233 | 0.259 | 0.311 |
| Azoarcus sp. BH72 | Betaproteobacteria | Rhodocyclaceae | 10 | 0.186 | 0.369 | 0.409 | 0.489 |
| Azospirillum sp. B510 | Alphaproteobacteria | Rhodospirillaceae | 25 | 0.094 | 0.238 | 0.264 | 0.317 |
| Azotobacter vinelandii DJ | Gammaproteobacteria | Pseudomonadaceae | 22 | 0.187 | 0.253 | 0.281 | 0.337 |
| Bartonella grahamii as4aup | Alphaproteobacteria | Bartonellaceae | 30 | 0.184 | 0.218 | 0.242 | 0.290 |
| Beijerinckia indica subsp. indica ATCC 9039 | Alphaproteobacteria | Beijerinckiaceae | 24 | 0.137 | 0.243 | 0.270 | 0.323 |
| Bifidobacterium dentium Bd1 | Actinobacteria | Bifidobacteriaceae | 34 | 0.119 | 0.205 | 0.228 | 0.273 |
| Brevundimonas subvibriodes | Alphaproteobacteria | Caulobacteraceae | 13 | 0.220 | 0.325 | 0.361 | 0.432 |
| Burkholderia phytofirmans PsJN | Betaproteobacteria | Burkholderiaceae | 23 | 0.102 | 0.248 | 0.275 | 0.330 |
| Caldicellulosiruptor hydrothermalis 108 | Firmicutes | Unknown | 21 | 0.111 | 0.259 | 0.288 | 0.345 |
| Calditerrivibrio nitroreducens DSM 19672 | Deferribacteres | Deferribacteraceae | 15 | 0.290 | 0.304 | 0.338 | 0.404 |
| Campylobacter hominis ATCC BAA-381 | Epsilonproteobacteria | Campylobacteraceae | 43 | 0.138 | 0.183 | 0.203 | 0.243 |
| Candidatus Accumulibacter phosphatis clade IIA str. UW-1 | Betaproteobacteria | Unknown | 29 | 0.244 | 0.221 | 0.246 | 0.295 |
| Candidatus Hamiltonella defensa 5AT (Acyrthosiphon pisum) | Gammaproteobacteria | Enterobacteriaceae | 18 | 0.147 | 0.279 | 0.309 | 0.371 |
| Candidatus Koribacter versatilis Ellin345 | Acidobacteria | Unknown | 26 | 0.135 | 0.233 | 0.259 | 0.311 |
| Candidatus Liberibacter asiaticus str. psy62 | Alphaproteobacteria | Rhizobiaceae | 29 | 0.069 | 0.221 | 0.246 | 0.295 |
| Candidatus Puniceispirillum marinum IMCC1322 | Alphaproteobacteria | Unknown | 28 | 0.122 | 0.225 | 0.250 | 0.300 |
| Candidatus Ruthia magnifica str. Cm (Calyptogena magnifica) | Gammaproteobacteria | Unknown | 33 | 0.277 | 0.208 | 0.231 | 0.277 |
| Candidatus Vesicomyosocius okutanii HA | Gammaproteobacteria | Unknown | 36 | 0.166 | 0.199 | 0.221 | 0.265 |
| Capnocytophaga ochracea DSM 7271 | Bacteroidetes | Flavobacteriaceae | 33 | 0.072 | 0.208 | 0.231 | 0.277 |
| Carboxydothermus hydrogenoformans Z-2901 | Firmicutes | Thermoanaerobacteraceae | 24 | 0.094 | 0.243 | 0.270 | 0.323 |
| Chlamydophila felis Fe/C-56 | Chlamydiae | Chlamydiaceae | 13 | 0.229 | 0.325 | 0.361 | 0.432 |
| Chlorobaculum parvum NCIB 8327 | Chlorobi | Chlorobiaceae | 21 | 0.205 | 0.259 | 0.288 | 0.345 |
| Chlorobium tepidum TLS | Chlorobi | Chlorobiaceae | 17 | 0.257 | 0.286 | 0.318 | 0.381 |
| Chloroherpeton thalassium ATCC 35110 | Chlorobi | Chlorobiaceae | 23 | 0.251 | 0.248 | 0.275 | 0.330 |
| Chromobacterium violaceum CV1192.gb | Betaproteobacteria | Neisseriaceae | 23 | 0.147 | 0.248 | 0.275 | 0.330 |
| Chromohalobacter salexigens DSM 3043 | Gammaproteobacteria | Halomonadaceae | 17 | 0.138 | 0.286 | 0.318 | 0.381 |
| Citrobacter koseri ATCC BAA-895 | Gammaproteobacteria | Enterobacteriaceae | 34 | 0.113 | 0.205 | 0.228 | 0.273 |
| Clostridiales genomosp. BVAB3 str. UPII9-5 | Firmicutes | Unknown | 18 | 0.091 | 0.279 | 0.309 | 0.371 |
| Clostridium acetobutylicum ATCC 824 | Firmicutes | Clostridiaceae | 22 | 0.093 | 0.253 | 0.281 | 0.337 |
| Colwellia psychrerythraea 34H | Gammaproteobacteria | Colwelliaceae | 39 | 0.207 | 0.192 | 0.213 | 0.255 |
| Conexibacter woesei DSM 14684 | Actinobacteria | Conexibacteraceae | 30 | 0.362 | 0.218 | 0.242 | 0.290 |
| Coraliomargarita akajimensis DSM 45221 | Verrucomicrobia | Puniceicoccaceae | 21 | 0.126 | 0.259 | 0.288 | 0.345 |
| Corynebacterium glutamicum ATCC 13032 | Actinobacteria | Corynebacteriaceae | 34 | 0.135 | 0.205 | 0.228 | 0.273 |
| Cronobacter turicensis z3032 | Gammaproteobacteria | Enterobacteriaceae | 29 | 0.082 | 0.221 | 0.246 | 0.295 |
| Cryptobacterium curtum DSM 15641 | Actinobacteria | Coriobacteriaceae | 11 | 0.105 | 0.352 | 0.391 | 0.468 |
| Cyanothece sp. ATCC 51142 | Cyanobacteria | Unknown | 43 | 0.106 | 0.183 | 0.203 | 0.243 |
| Deinococcus radiodurans R1 | Deinococcus-Thermus | Deinococcaceae | 25 | 0.091 | 0.238 | 0.264 | 0.317 |
| Desulfarculus baarsii DSM 2075 | Deltaproteobacteria | Desulfarculaceae | 18 | 0.198 | 0.279 | 0.309 | 0.371 |
| Desulfitobacterium hafniense DCB-2 | Firmicutes | Peptococcaceae | 22 | 0.228 | 0.253 | 0.281 | 0.337 |
| Desulfohalobium retbaense DSM 5692 | Deltaproteobacteria | Desulfohalobiaceae | 23 | 0.250 | 0.248 | 0.275 | 0.330 |
| Desulfotalea psychrophila LSv54 | Deltaproteobacteria | Desulfobulbaceae | 27 | 0.079 | 0.229 | 0.255 | 0.305 |
| Desulfovibrio magneticus RS-1 | Deltaproteobacteria | Desulfovibrionaceae | 36 | 0.051 | 0.199 | 0.221 | 0.265 |
| Desulfurivibrio alkaliphilus AHT2 | Deltaproteobacteria | Desulfobulbaceae | 17 | 0.147 | 0.286 | 0.318 | 0.381 |
| Dichelobacter nodosus VCS1703A | Gammaproteobacteria | Cardiobacteriaceae | 34 | 0.110 | 0.205 | 0.228 | 0.273 |
| Dickeya dadantii 3937 | Gammaproteobacteria | Enterobacteriaceae | 33 | 0.089 | 0.208 | 0.231 | 0.277 |
| Dinoroseobacter shibae DFL 12 | Alphaproteobacteria | Rhodobacteraceae | 25 | 0.259 | 0.238 | 0.264 | 0.317 |
| Dyadobacter fermentans DSM 18053 | Bacteroidetes | Cytophagaceae | 40 | 0.070 | 0.189 | 0.210 | 0.252 |
| Edwardsiella ictaluri 93-146 | Gammaproteobacteria | Enterobacteriaceae | 21 | 0.141 | 0.259 | 0.288 | 0.345 |
| Enterobacter sp. 638 | Gammaproteobacteria | Enterobacteriaceae | 35 | 0.112 | 0.202 | 0.224 | 0.269 |
| Enterococcus faecalis V583 | Firmicutes | Enterococcaceae | 41 | 0.114 | 0.187 | 0.208 | 0.249 |
| Erwinia billingiae Eb661 | Gammaproteobacteria | Enterobacteriaceae | 38 | 0.216 | 0.194 | 0.216 | 0.258 |
| Escherichia coli K-12 substr. DH10B | Gammaproteobacteria | Enterobacteriaceae | 27 | 0.073 | 0.229 | 0.255 | 0.305 |
| Eubacterium eligens ATCC 27750 | Firmicutes | Eubacteriaceae | 14 | 0.345 | 0.314 | 0.349 | 0.418 |
| Ferrimonas balearica DSM 9799 | Gammaproteobacteria | Ferrimonadaceae | 30 | 0.123 | 0.218 | 0.242 | 0.290 |
| Fervidobacterium nodosum Rt17-B1 | Thermotogae | Thermotogaceae | 20 | 0.175 | 0.265 | 0.294 | 0.352 |
| Finegoldia magna ATCC 29328 | Firmicutes | Unknown | 23 | 0.096 | 0.248 | 0.275 | 0.330 |
| Flavobacterium psychrophilum JIP02/86 | Bacteroidetes | Flavobacteriaceae | 41 | 0.118 | 0.187 | 0.208 | 0.249 |
| Francisella tularensis subsp. tularensis FSC198 | Gammaproteobacteria | Francisellaceae | 11 | 0.136 | 0.352 | 0.391 | 0.468 |
| Frankia sp. EAN1pec | Actinobacteria | Frankiaceae | 29 | 0.259 | 0.221 | 0.246 | 0.295 |
| Fusobacterium nucleatum subsp. nucleatum ATCC 25586 | Fusobacteria | Fusobacteriaceae | 16 | 0.208 | 0.295 | 0.327 | 0.392 |
| Gallionella capsiferriformans ES-2 | Betaproteobacteria | Gallionellaceae | 19 | 0.147 | 0.271 | 0.301 | 0.361 |
| gamma proteobacterium HdN1 | Gammaproteobacteria | Unknown | 33 | 0.096 | 0.208 | 0.231 | 0.277 |
| Gardnerella vaginalis 409-05 | Actinobacteria | Bifidobacteriaceae | 20 | 0.114 | 0.265 | 0.294 | 0.352 |
| Geobacillus sp. Y412MC10 | Firmicutes | Bacillaceae | 32 | 0.098 | 0.211 | 0.234 | 0.281 |
| Geobacter sp. FRC-32 | Deltaproteobacteria | Geobacteraceae | 24 | 0.135 | 0.243 | 0.270 | 0.323 |
| Gordonia bronchialis DSM 43247 | Actinobacteria | Gordoniaceae | 23 | 0.217 | 0.248 | 0.275 | 0.330 |
| Haemophilus ducreyi 35000HP | Gammaproteobacteria | Pasteurellaceae | 39 | 0.144 | 0.192 | 0.213 | 0.255 |
| Haliangium ochraceum DSM 14365 | Deltaproteobacteria | Haliangiaceae | 34 | 0.144 | 0.205 | 0.228 | 0.273 |
| Halomonas elongata DSM 2581 | Gammaproteobacteria | Halomonadaceae | 13 | 0.243 | 0.325 | 0.361 | 0.432 |
| Halorhodospira halophila SL1 | Gammaproteobacteria | Ectothiorhodospiraceae | 20 | 0.104 | 0.265 | 0.294 | 0.352 |
| Halothermothrix orenii H 168 | Firmicutes | Halanaerobiaceae | 31 | 0.275 | 0.214 | 0.238 | 0.285 |
| Halothiobacillus neapolitanus c2 | Gammaproteobacteria | Halothiobacillaceae | 31 | 0.140 | 0.214 | 0.238 | 0.285 |
| Helicobacter pylori 26695 | Epsilonproteobacteria | Helicobacteraceae | 43 | 0.170 | 0.183 | 0.203 | 0.243 |
| Heliobacterium modesticaldum Ice1 | Firmicutes | Heliobacteriaceae | 26 | 0.147 | 0.233 | 0.259 | 0.311 |
| Herbaspirillum seropedicae SmR1 | Betaproteobacteria | Oxalobacteraceae | 11 | 0.114 | 0.352 | 0.391 | 0.468 |
| Herminiimonas arsenicoxydans | Betaproteobacteria | Oxalobacteraceae | 16 | 0.207 | 0.295 | 0.327 | 0.392 |
| Herpetosiphon aurantiacus ATCC 23779 | Chloroflexi | Herpetosiphonaceae | 37 | 0.139 | 0.197 | 0.218 | 0.262 |
| Hirschia baltica ATCC 49814 | Alphaproteobacteria | Hyphomonadaceae | 34 | 0.218 | 0.205 | 0.228 | 0.273 |
| Idiomarina loihiensis L2TR | Gammaproteobacteria | Idiomarinaceae | 30 | 0.119 | 0.218 | 0.242 | 0.290 |
| Jannaschia sp. CCS1 | Alphaproteobacteria | Rhodobacteraceae | 33 | 0.279 | 0.208 | 0.231 | 0.277 |
| Jonesia denitrificans DSM 20603 | Actinobacteria | Jonesiaceae | 33 | 0.151 | 0.208 | 0.231 | 0.277 |
| Ketogulonicigenium vulgare Y25 | Alphaproteobacteria | Rhodobacteraceae | 35 | 0.054 | 0.202 | 0.224 | 0.269 |
| Kineococcus radiotolerans SRS30216 | Actinobacteria | Kineosporiaceae | 29 | 0.152 | 0.221 | 0.246 | 0.295 |
| Klebsiella pneumoniae 342 | Gammaproteobacteria | Enterobacteriaceae | 36 | 0.215 | 0.199 | 0.221 | 0.265 |
| Kosmotoga olearia TBF 19.5.1 | Thermotogae | Thermotogaceae | 18 | 0.196 | 0.279 | 0.309 | 0.371 |
| Kribbella flavida DSM 17836 | Actinobacteria | Nocardioidaceae | 21 | 0.219 | 0.259 | 0.288 | 0.345 |
| Lactobacillus brevis ATCC 367 | Firmicutes | Lactobacillaceae | 31 | 0.085 | 0.214 | 0.238 | 0.285 |
| Lactococcus lactis subsp. lactis Il1403 | Firmicutes | Streptococcaceae | 30 | 0.107 | 0.218 | 0.242 | 0.290 |
| Laribacter hongkongensis HLHK9 | Betaproteobacteria | Neisseriaceae | 28 | 0.170 | 0.225 | 0.250 | 0.300 |
| Leadbetterella byssophila DSM 17132 | Bacteroidetes | Cytophagaceae | 27 | 0.124 | 0.229 | 0.255 | 0.305 |
| Legionella pneumophila 2300/99 Alcoy | Gammaproteobacteria | Legionellaceae | 30 | 0.084 | 0.218 | 0.242 | 0.290 |
| Leptothrix cholodnii SP-6 | Betaproteobacteria | Unknown | 29 | 0.213 | 0.221 | 0.246 | 0.295 |
| Leuconostoc citreum KM20 | Firmicutes | Unknown | 32 | 0.108 | 0.211 | 0.234 | 0.281 |
| Listeria monocytogenes 08-5923 | Firmicutes | Listeriaceae | 31 | 0.069 | 0.214 | 0.238 | 0.285 |
| Lysinibacillus sphaericus C3-41 | Firmicutes | Bacillaceae | 20 | 0.084 | 0.265 | 0.294 | 0.352 |
| Magnetococcus sp. MC-1 | Proteobacteria | Unknown | 40 | 0.299 | 0.189 | 0.210 | 0.252 |
| Magnetospirillum magneticum AMB-1 | Alphaproteobacteria | Rhodospirillaceae | 25 | 0.133 | 0.238 | 0.264 | 0.317 |
| Mannheimia succiniciproducens MBEL55E | Gammaproteobacteria | Pasteurellaceae | 26 | 0.158 | 0.233 | 0.259 | 0.311 |
| Marinobacter aquaeolei VT8 | Gammaproteobacteria | Alteromonadaceae | 24 | 0.163 | 0.243 | 0.270 | 0.323 |
| Marinomonas sp. MWYL1 | Gammaproteobacteria | Unknown | 39 | 0.207 | 0.192 | 0.213 | 0.255 |
| Methylobacterium radiotolerans JCM 2831 | Alphaproteobacteria | Methylobacteriaceae | 35 | 0.216 | 0.202 | 0.224 | 0.269 |
| Methylotenera sp. 301 | Betaproteobacteria | Methylophilaceae | 31 | 0.224 | 0.214 | 0.238 | 0.285 |
| Microcystis aeruginosa NIES-843 | Cyanobacteria | Unknown | 24 | 0.117 | 0.243 | 0.270 | 0.323 |
| Mobiluncus curtisii ATCC 43063 | Actinobacteria | Actinomycetaceae | 36 | 0.282 | 0.199 | 0.221 | 0.265 |
| Moraxella catarrhalis RH4 | Gammaproteobacteria | Moraxellaceae | 35 | 0.088 | 0.202 | 0.224 | 0.269 |
| Mycoplasma genitalium G37 | Tenericutes | Mycoplasmataceae | 36 | 0.101 | 0.199 | 0.221 | 0.265 |
| Nakamurella multipartita DSM 44233 | Actinobacteria | Nakamurellaceae | 24 | 0.197 | 0.243 | 0.270 | 0.323 |
| Natranaerobius thermophilus JW/NM-WN-LF | Firmicutes | Natranaerobiaceae | 19 | 0.101 | 0.271 | 0.301 | 0.361 |
| Nitratiruptor sp. SB155-2 | Epsilonproteobacteria | Unknown | 36 | 0.119 | 0.199 | 0.221 | 0.265 |
| Nitrosococcus halophilus Nc4 | Gammaproteobacteria | Chromatiaceae | 30 | 0.131 | 0.218 | 0.242 | 0.290 |
| Nocardioides sp. JS614 | Actinobacteria | Nocardioidaceae | 26 | 0.223 | 0.233 | 0.259 | 0.311 |
| Nostoc azollae' 0708 | Cyanobacteria | Nostocaceae | 24 | 0.153 | 0.243 | 0.270 | 0.323 |
| Paenibacillus polymyxa E681 | Firmicutes | Paenibacillaceae | 32 | 0.110 | 0.211 | 0.234 | 0.281 |
| Pantoea ananatis LMG 20103 | Gammaproteobacteria | Enterobacteriaceae | 33 | 0.116 | 0.208 | 0.231 | 0.277 |
| Pasteurella multocida subsp. multocida str. Pm70 | Gammaproteobacteria | Pasteurellaceae | 33 | 0.211 | 0.208 | 0.231 | 0.277 |
| Pectobacterium atrosepticum SCRI1043 | Gammaproteobacteria | Enterobacteriaceae | 37 | 0.109 | 0.197 | 0.218 | 0.262 |
| Pediococcus pentosaceus ATCC 25745 | Firmicutes | Lactobacillaceae | 27 | 0.150 | 0.229 | 0.255 | 0.305 |
| Pelodictyon phaeoclathratiforme BU-1 | Chlorobi | Chlorobiaceae | 15 | 0.189 | 0.304 | 0.338 | 0.404 |
| Petrotoga mobilis SJ95 | Thermotogae | Thermotogaceae | 24 | 0.129 | 0.243 | 0.270 | 0.323 |
| Photorhabdus asymbiotica | Gammaproteobacteria | Enterobacteriaceae | 24 | 0.070 | 0.243 | 0.270 | 0.323 |
| Polaromonas naphthalenivorans CJ2 | Betaproteobacteria | Comamonadaceae | 13 | 0.095 | 0.325 | 0.361 | 0.432 |
| Prevotella melaninogenica ATCC 25845 | Bacteroidetes | Prevotellaceae | 41 | 0.096 | 0.187 | 0.208 | 0.249 |
| Prochlorococcus marinus str. MIT 9313 | Cyanobacteria | Prochlorococcaceae | 16 | 0.216 | 0.295 | 0.327 | 0.392 |
| Propionibacterium acnes KPA171202 | Actinobacteria | Propionibacteriaceae | 26 | 0.214 | 0.233 | 0.259 | 0.311 |
| Proteus mirabilis HI4320 | Gammaproteobacteria | Enterobacteriaceae | 33 | 0.144 | 0.208 | 0.231 | 0.277 |
| Pseudoalteromonas atlantica T6c | Gammaproteobacteria | Pseudoalteromonadaceae | 37 | 0.120 | 0.197 | 0.218 | 0.262 |
| Pseudomonas fluorescens Pf0-1 | Gammaproteobacteria | Pseudomonadaceae | 27 | 0.110 | 0.229 | 0.255 | 0.305 |
| Psychrobacter sp. PRwf-1 | Gammaproteobacteria | Moraxellaceae | 38 | 0.221 | 0.194 | 0.216 | 0.258 |
| Psychromonas ingrahamii 37 | Gammaproteobacteria | Psychromonadaceae | 38 | 0.222 | 0.194 | 0.216 | 0.258 |
| Renibacterium salmoninarum ATCC 33209 | Actinobacteria | Micrococcaceae | 27 | 0.100 | 0.229 | 0.255 | 0.305 |
| Rhodobacter capsulatus SB 1003 | Alphaproteobacteria | Rhodobacteraceae | 38 | 0.053 | 0.194 | 0.216 | 0.258 |
| Rhodopirellula baltica SH 1 | Planctomycetes | Planctomycetaceae | 31 | 0.270 | 0.214 | 0.238 | 0.285 |
| Rhodopseudomonas palustris TIE-1 | Alphaproteobacteria | Bradyrhizobiaceae | 17 | 0.126 | 0.286 | 0.318 | 0.381 |
| Rhodospirillum rubrum ATCC 11170 | Alphaproteobacteria | Rhodospirillaceae | 36 | 0.127 | 0.199 | 0.221 | 0.265 |
| Rhodothermus marinus DSM 4252 | Bacteroidetes | Rhodothermaceae | 20 | 0.136 | 0.265 | 0.294 | 0.352 |
| Riemerella anatipestifer DSM 15868 | Bacteroidetes | Flavobacteriaceae | 29 | 0.176 | 0.221 | 0.246 | 0.295 |
| Roseiflexus sp. RS-1 | Chloroflexi | Chloroflexaceae | 15 | 0.187 | 0.304 | 0.338 | 0.404 |
| Roseobacter denitrificans OCh 114 | Alphaproteobacteria | Rhodobacteraceae | 32 | 0.197 | 0.211 | 0.234 | 0.281 |
| Rothia dentocariosa ATCC 17931 | Actinobacteria | Micrococcaceae | 34 | 0.171 | 0.205 | 0.228 | 0.273 |
| Saccharomonospora viridis DSM 43017 | Actinobacteria | Pseudonocardiaceae | 22 | 0.169 | 0.253 | 0.281 | 0.337 |
| Saccharophagus degradans 2-40 | Gammaproteobacteria | Alteromonadaceae | 27 | 0.160 | 0.229 | 0.255 | 0.305 |
| Salinispora arenicola CNS-205 | Actinobacteria | Micromonosporaceae | 27 | 0.120 | 0.229 | 0.255 | 0.305 |
| Salmonella enterica subsp. enterica serovar Dublin str. CT_02021853 | Gammaproteobacteria | Enterobacteriaceae | 32 | 0.154 | 0.211 | 0.234 | 0.281 |
| Segniliparus rotundus DSM 44985 | Actinobacteria | Segniliparaceae | 42 | 0.076 | 0.185 | 0.205 | 0.246 |
| Serratia proteamaculans 568 | Gammaproteobacteria | Enterobacteriaceae | 37 | 0.175 | 0.197 | 0.218 | 0.262 |
| Shewanella baltica OS155 | Gammaproteobacteria | Shewanellaceae | 41 | 0.180 | 0.187 | 0.208 | 0.249 |
| Silicibacter sp. TM1040 | Alphaproteobacteria | Rhodobacteraceae | 26 | 0.087 | 0.233 | 0.259 | 0.311 |
| Sodalis glossinidius str. 'morsitans' | Gammaproteobacteria | Enterobacteriaceae | 27 | 0.140 | 0.229 | 0.255 | 0.305 |
| Solibacter usitatus Ellin6076 | Acidobacteria | Solibacteraceae | 21 | 0.113 | 0.259 | 0.288 | 0.345 |
| Sorangium cellulosum 'So ce 56' | Deltaproteobacteria | Polyangiaceae | 38 | 0.118 | 0.194 | 0.216 | 0.258 |
| Sphingomonas wittichii RW1 | Alphaproteobacteria | Sphingomonadaceae | 12 | 0.167 | 0.338 | 0.375 | 0.449 |
| Spirochaeta smaragdinae DSM 11293 | Spirochaetes | Spirochaetaceae | 26 | 0.116 | 0.233 | 0.259 | 0.311 |
| Spirosoma linguale DSM 74 | Bacteroidetes | Cytophagaceae | 39 | 0.169 | 0.192 | 0.213 | 0.255 |
| Stenotrophomonas maltophilia K279a | Gammaproteobacteria | Xanthomonadaceae | 20 | 0.151 | 0.265 | 0.294 | 0.352 |
| Streptococcus gallolyticus UCN34 | Firmicutes | Streptococcaceae | 36 | 0.119 | 0.199 | 0.221 | 0.265 |
| Sulfuricurvum kujiense DSM 16994 | Epsilonproteobacteria | Helicobacteraceae | 30 | 0.210 | 0.218 | 0.242 | 0.290 |
| Sulfurimonas denitrificans DSM 1251 | Epsilonproteobacteria | Helicobacteraceae | 36 | 0.158 | 0.199 | 0.221 | 0.265 |
| Sulfurospirillum deleyianum DSM 6946 | Epsilonproteobacteria | Campylobacteraceae | 45 | 0.235 | 0.179 | 0.198 | 0.238 |
| Synechococcus sp. PCC 7002 | Cyanobacteria | Unknown | 36 | 0.112 | 0.199 | 0.221 | 0.265 |
| Synechocystis sp. PCC 6803 | Cyanobacteria | Unknown | 32 | 0.189 | 0.211 | 0.234 | 0.281 |
| Teredinibacter turnerae T7901 | Gammaproteobacteria | Unknown | 37 | 0.177 | 0.197 | 0.218 | 0.262 |
| Thauera sp. MZ1T | Betaproteobacteria | Rhodocyclaceae | 11 | 0.105 | 0.352 | 0.391 | 0.468 |
| Thermoanaerobacter mathranii subsp. mathranii str. A3 | Firmicutes | Thermoanaerobacteraceae | 21 | 0.142 | 0.259 | 0.288 | 0.345 |
| Thermocrinis albus DSM 14484 | Aquificae | Aquificaceae | 23 | 0.105 | 0.248 | 0.275 | 0.330 |
| Thermomonospora curvata DSM 43183 | Actinobacteria | Thermomonosporaceae | 31 | 0.210 | 0.214 | 0.238 | 0.285 |
| Thermosipho melanesiensis BI429 | Thermotogae | Thermotogaceae | 27 | 0.081 | 0.229 | 0.255 | 0.305 |
| Thermosynechococcus elongatus BP-1 | Cyanobacteria | Unknown | 27 | 0.144 | 0.229 | 0.255 | 0.305 |
| Thermotoga sp. RQ2 | Thermotogae | Thermotogaceae | 35 | 0.095 | 0.202 | 0.224 | 0.269 |
| Thioalkalivibrio sp. K90mix | Gammaproteobacteria | Ectothiorhodospiraceae | 12 | 0.293 | 0.338 | 0.375 | 0.449 |
| Thiomicrospira crunogena XCL-2 | Gammaproteobacteria | Piscirickettsiaceae | 42 | 0.090 | 0.185 | 0.205 | 0.246 |
| Thiomonas intermedia K12 | Betaproteobacteria | Unknown | 20 | 0.092 | 0.265 | 0.294 | 0.352 |
| Trichodesmium erythraeum IMS101 | Cyanobacteria | Unknown | 42 | 0.109 | 0.185 | 0.205 | 0.246 |
| Truepera radiovictrix DSM 17093 | Deinococcus-Thermus | Trueperaceae | 38 | 0.120 | 0.194 | 0.216 | 0.258 |
| Tsukamurella paurometabola DSM 20162 | Actinobacteria | Tsukamurellaceae | 18 | 0.154 | 0.279 | 0.309 | 0.371 |
| Veillonella parvula DSM 2008 | Firmicutes | Veillonellaceae | 24 | 0.119 | 0.243 | 0.270 | 0.323 |
| Verminephrobacter eiseniae EF01-2 | Betaproteobacteria | Comamonadaceae | 17 | 0.164 | 0.286 | 0.318 | 0.381 |
| Vibrio cholerae O1 biovar eltor str. N16961 | Gammaproteobacteria | Vibrionaceae | 37 | 0.128 | 0.197 | 0.218 | 0.262 |
| Waddlia chondrophila WSU 86-1044 | Chlamydiae | Waddliaceae | 24 | 0.187 | 0.243 | 0.270 | 0.323 |
| Wolinella succinogenes DSM 1740 | Epsilonproteobacteria | Helicobacteraceae | 40 | 0.284 | 0.189 | 0.210 | 0.252 |
| Xanthomonas albilineans | Gammaproteobacteria | Xanthomonadaceae | 16 | 0.170 | 0.295 | 0.327 | 0.392 |
| Xenorhabdus nematophila ATCC 19061 | Gammaproteobacteria | Enterobacteriaceae | 20 | 0.112 | 0.265 | 0.294 | 0.352 |
| Xylanimonas cellulosilytica DSM 15894 | Actinobacteria | Promicromonosporaceae | 20 | 0.212 | 0.265 | 0.294 | 0.352 |
| Xylella fastidiosa 9a5c | Gammaproteobacteria | Xanthomonadaceae | 14 | 0.147 | 0.314 | 0.349 | 0.418 |
| Yersinia enterocolitica subsp. enterocolitica 8081 | Gammaproteobacteria | Enterobacteriaceae | 35 | 0.171 | 0.202 | 0.224 | 0.269 |
| Zymomonas mobilis subsp. mobilis NCIMB 11163 | Alphaproteobacteria | Sphingomonadaceae | 28 | 0.089 | 0.225 | 0.250 | 0.300 |
