# Supplementary material for: Selection for ancient periodic motifs that do not impart DNA bending
Source: PLoS Genet. 2020 Oct 6;16(10):e1009042. doi: 10.1371/journal.pgen.1009042 (PMC7537859; doi:10.1371/journal.pgen.1009042)
Supplement: S5 Table — (DOCX) [file pgen.1009042.s005.docx]

**S5 Table**. Comparison of periodicity between genomes of different degrees of relatedness, normalized by difference in GC content.

| **Absolute value of difference in period** | | | | | |
| --- | --- | --- | --- | --- | --- |
|  |  | **Different Domains** | **Different Divisions** | **Different Families** | **Different Genera** |
| Archaea | Median | 0.59 | 0.8 | 0.22 | 0.145 |
|  | Upper Median | 0.92 | 1.14 | 0.55 | 0.33 |
|  | Lower Median | 0.3 | 0.41 | 0.09 | 0.085 |
|  | Count | 7845 | 73 | 85 | 56 |
|  | Median Delta GC | 2.36 | 2.36 | 2.37 | 2.385 |
|  | StDev (DeltaGC) | 1.37 | 1.17 | 1.58 | 1.53 |
|  |  |  |  |  |  |
| Other Bacteria | Median | 0.55 | 0.41 | 0.34 | 0.27 |
|  | Upper Median | 0.9 | 0.7 | 0.59 | 0.46 |
|  | Lower Median | 0.28 | 0.19 | 0.14 | 0.12 |
|  | Count | 3895 | 10915 | 2808 | 156 |
|  | Median Delta GC | 2.73 | 2.73 | 2.73 | 2.735 |
|  | StDev (DeltaGC) | 1.62 | 1.57 | 1.71 | 1.65 |
|  |  |  |  |  |  |
| Gammaproteobacteria | Median | 0.76 | 0.32 | 0.15 | 0.11 |
|  | Upper Median | 1.03 | 0.58 | 0.27 | 0.22 |
|  | Lower Median | 0.39 | 0.14 | 0.06 | 0.04 |
|  | Count | 1068 | 8195 | 1515 | 236 |
|  | Median Delta GC | 2.23 | 2.23 | 2.23 | 2.23 |
|  | StDev (DeltaGC) | 1.21 | 1.28 | 1.39 | 1.24 |
|  |  |  |  |  |  |
| **Pearson R of Strength of Individual Dinucleotide Periodicity** | | | | | |
|  | | | | | |
|  |  | **Different Domains** | **Different Divisions** | **Different Families** | **Different Genera** |
| Archaea | Median | 0.488 | 0.3372 | 0.6134 | 0.8159 |
|  | Upper Median | 0.6132 | 0.5 | 0.6994 | 0.8625 |
|  | Lower Median | 0.31445 | 0.19205 | 0.42045 | 0.74775 |
|  |  |  |  |  |  |
| Other Bacteria | Median | 0.4844 | 0.502 | 0.58235 | 0.72645 |
|  | Upper Median | 0.6117 | 0.6378 | 0.71105 | 0.80875 |
|  | Lower Median | 0.3192 | 0.3286 | 0.44335 | 0.619 |
|  |  |  |  |  |  |
| Gammaproteobacteria | Median | 0.5115 | 0.5714 | 0.6928 | 0.84545 |
|  | Upper Median | 0.6264 | 0.70165 | 0.7998 | 0.8913 |
|  | Lower Median | 0.2909 | 0.3756 | 0.5756 | 0.78095 |
