# Supplementary figures and images for: Selection for ancient periodic motifs that do not impart DNA bending
Source: PLoS Genet. 2020 Oct 6;16(10):e1009042. doi: 10.1371/journal.pgen.1009042 (PMC7537859; doi:10.1371/journal.pgen.1009042)

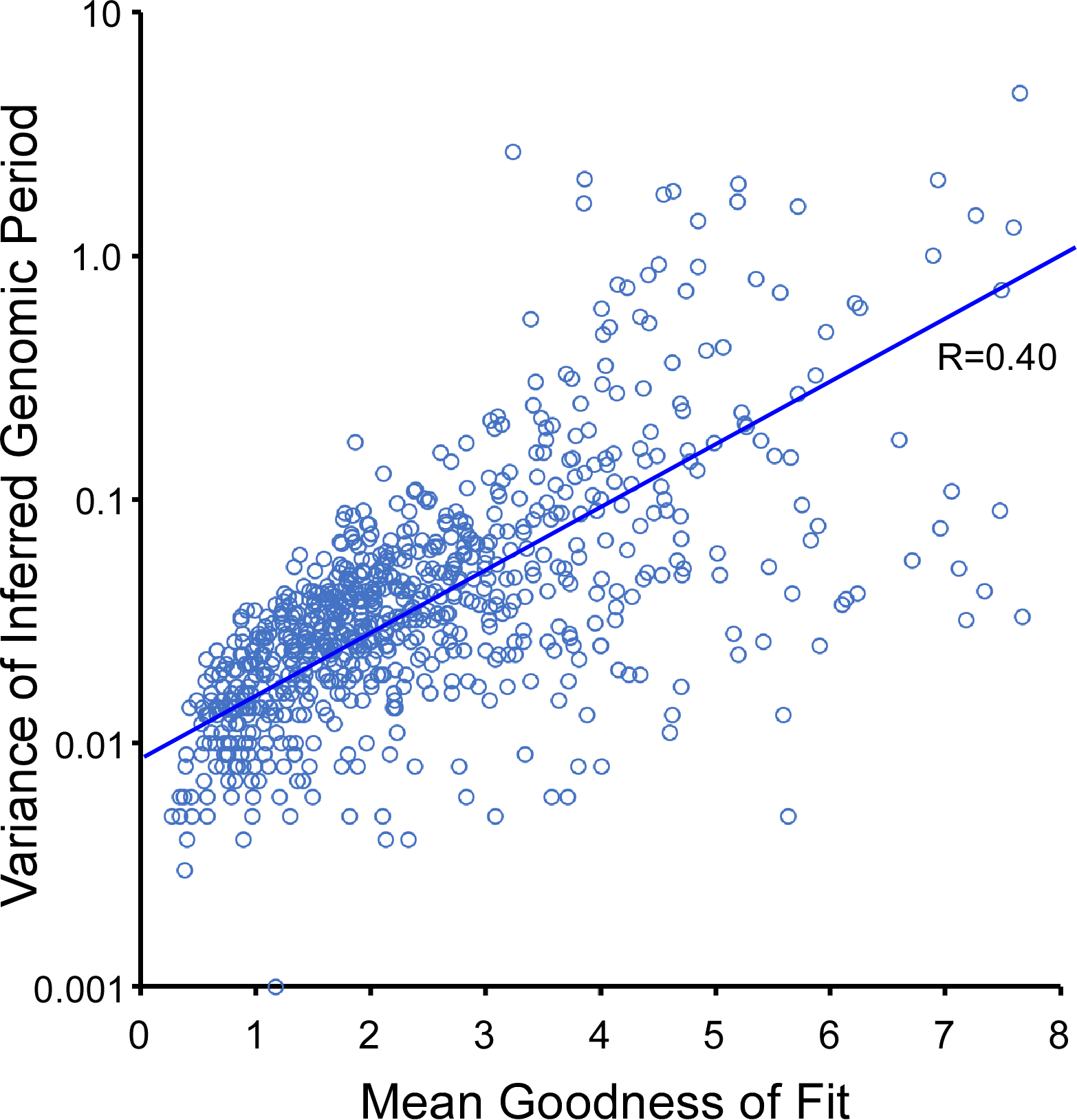

Supplement: S1 Fig — The goodness of fit to the curve fit to autocorrelation data was calculated for 764 genomes, none with 16S rRNA similarity greater than 97%. The variance in the genomic period was estimated by 1000 bootstrap resamples of the dinucleotide spacings. (TIF) [file pgen.1009042.s006.tif]

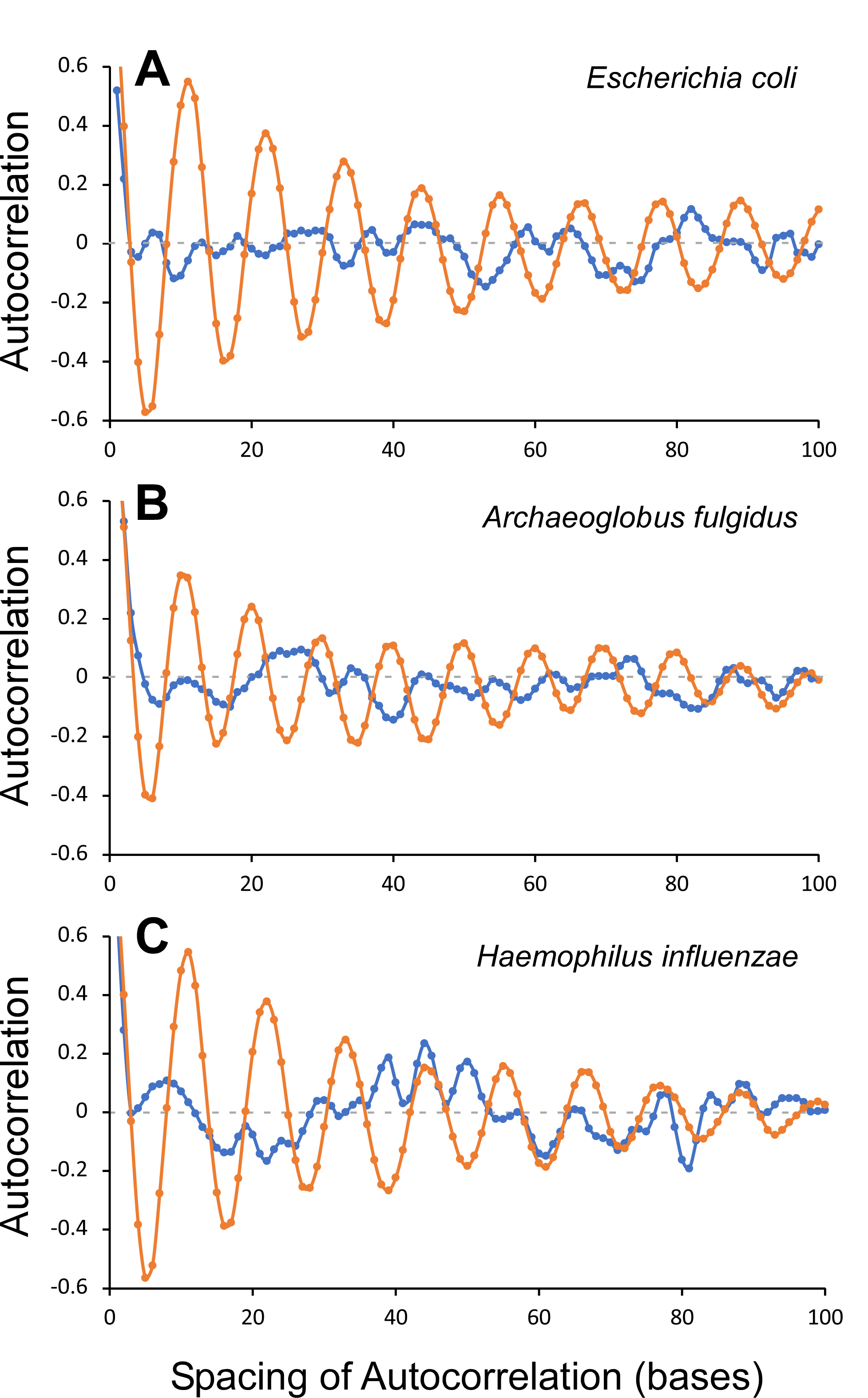

Supplement: S2 Fig — Autocorrelations of the abundances of spacings from both the genuine genome (orange) and from an artificial genome with the same codon-position-specific dinucleotide, trinucleotide and tetranucleotide frequencies, genes length distribution, and strand bias (blue) are shown for (A) E. coli, (B) A. fulgidus, and (C) H. influenzae. (TIF) [file pgen.1009042.s007.tif]

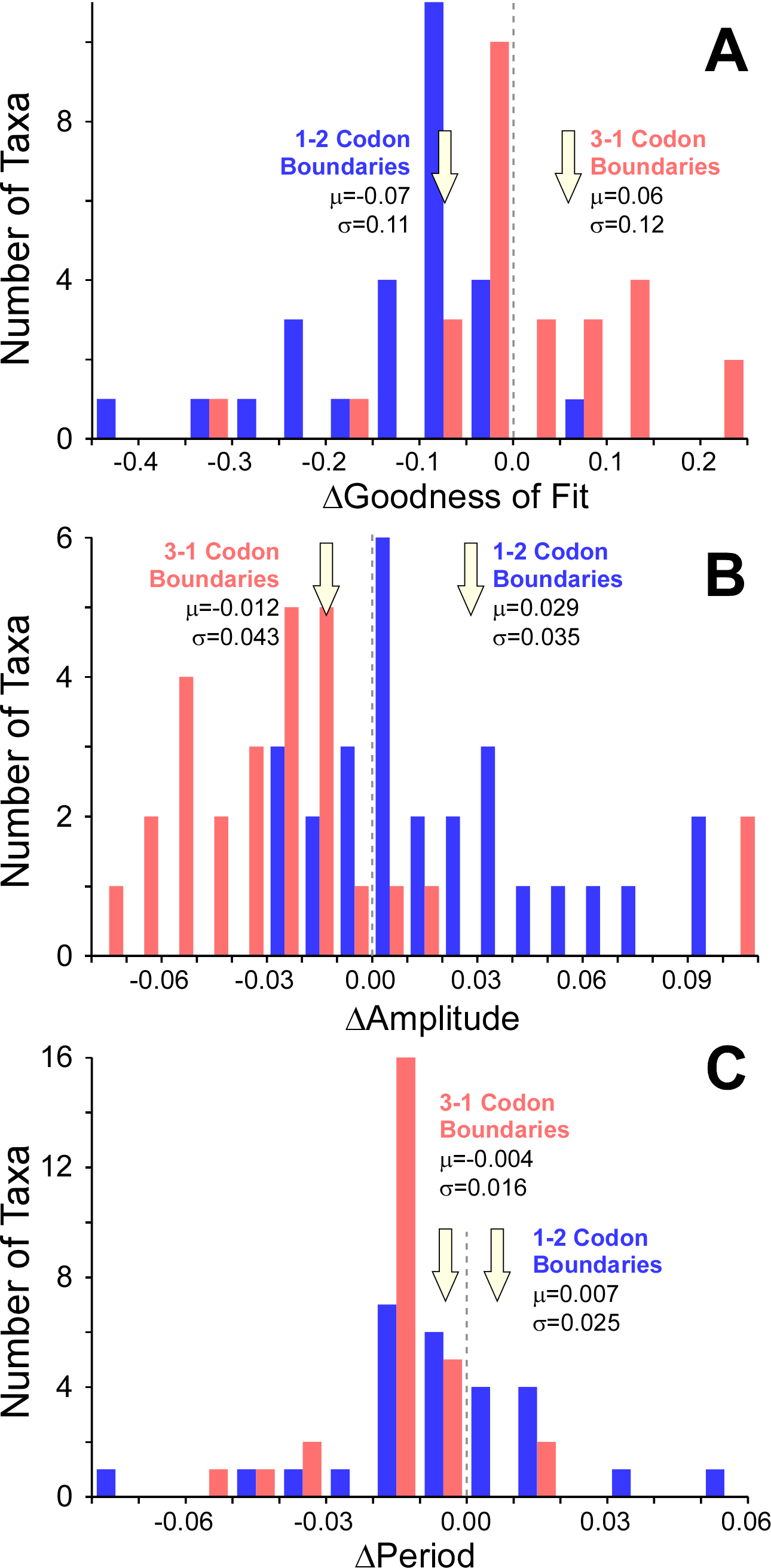

Supplement: S3 Fig — Autocorrelations of dinucleotide spacings were calculated for 27 genomes using all spacings (set I), or calculated by omitting either spacings between pairs of dinucleotides at the first and second codon positions within the same genes (set II), or spacings between pairs of dinucleotides at the third and first codon positions within the same genes (set III). Damped sine curves were fit to all three data sets. (A) Differences in goodness of fit between sets I and II (blue) or I and III (red). (B) Differences in amplitude between sets I and II (blue) or I and III (red). (C) Differences in period between sets I and II (blue) or I and III (red). The mean (μ) and standard deviation (σ) of the distributions of differences for the 27 genomes are displayed. (TIF) [file pgen.1009042.s008.tif]

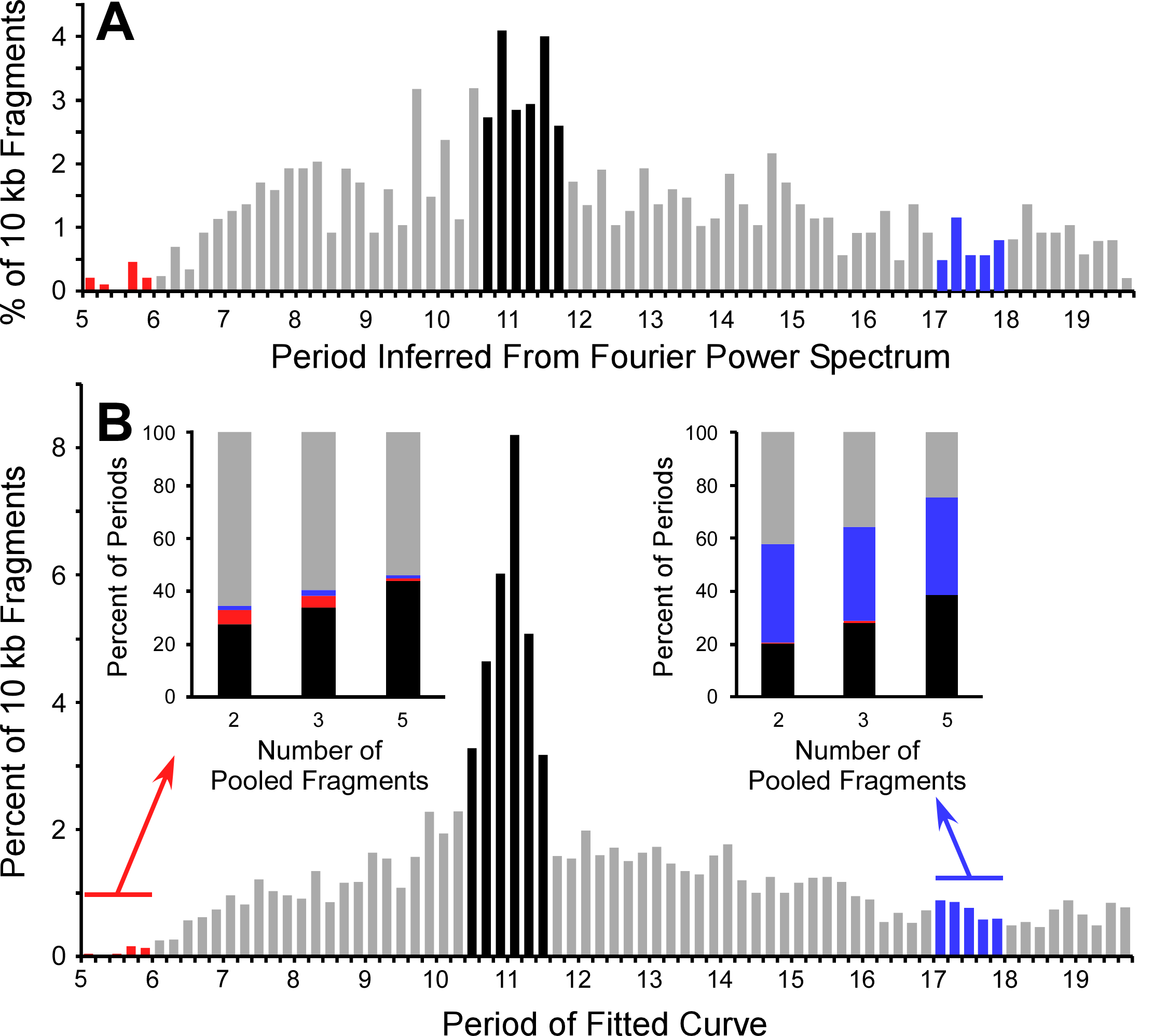

Supplement: S4 Fig — (A) Periods of sequential 10 kb fragments from the E. coli K12 MG1655 genome, stepping 5 kb, estimated from the power spectrum of Fourier transformation; data are replotted from Mrázek [22] Fig 2B, combining data from 0.1 bp intervals to 0.2 bp intervals. Fragments within 0.6 bp of the genomic period (11.05 bp) are shaded black. (B) Distribution of periods for best-fit curves of the autocorrelations of dinucleotide spacings of 270,000 random 10 kb fragments of the E. coli K12 MG1655 genome. Fragments within 0.6 bp of the genomic period (11.05 bp) are shaded black, fragments with periods estimated in the lower (5.0–6.0 bp) and higher (17.0–18.0 bp) ranges are shown in red and blue, respectively. The insets show percentages of estimated periods for sequences generated by pooling 2, 3, or 5 fragments initially showing periods within the lower (left inset) or higher (right inset) range of explored periods. Bars in the insets are color-coded to the ranges denoted in the primary figure. (TIF) [file pgen.1009042.s009.tif]

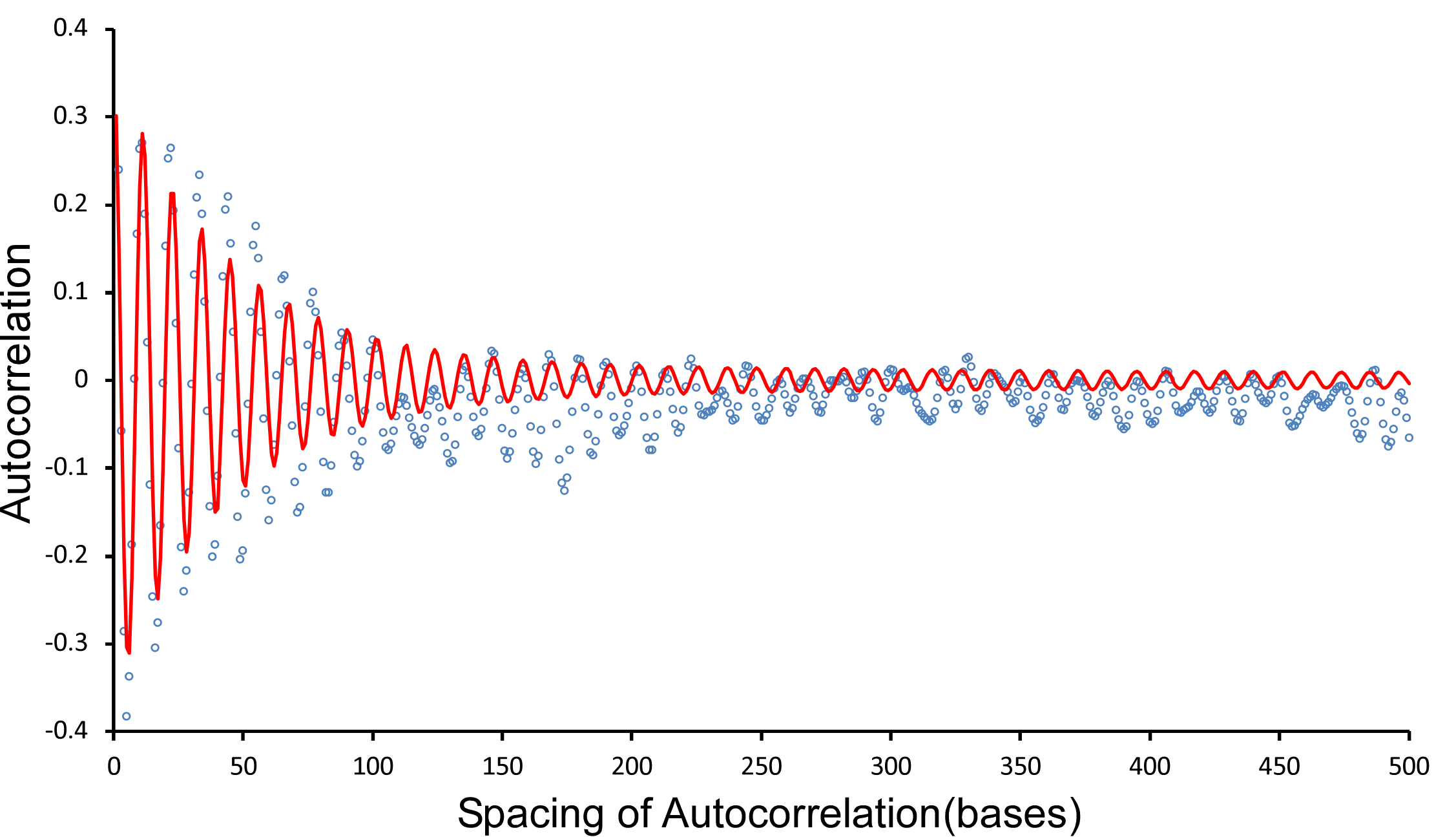

Supplement: S5 Fig — Autocorrelation of the abundances of spacings between cognate dinucleotides. Spacings were measured up to 1 kb apart; autocorrelations were calculated for differences in spacings up to 500 bp (see methods). Data are shown in blue and the best fit of a damped sine curve fit to these data is shown in red. (TIF) [file pgen.1009042.s010.tif]

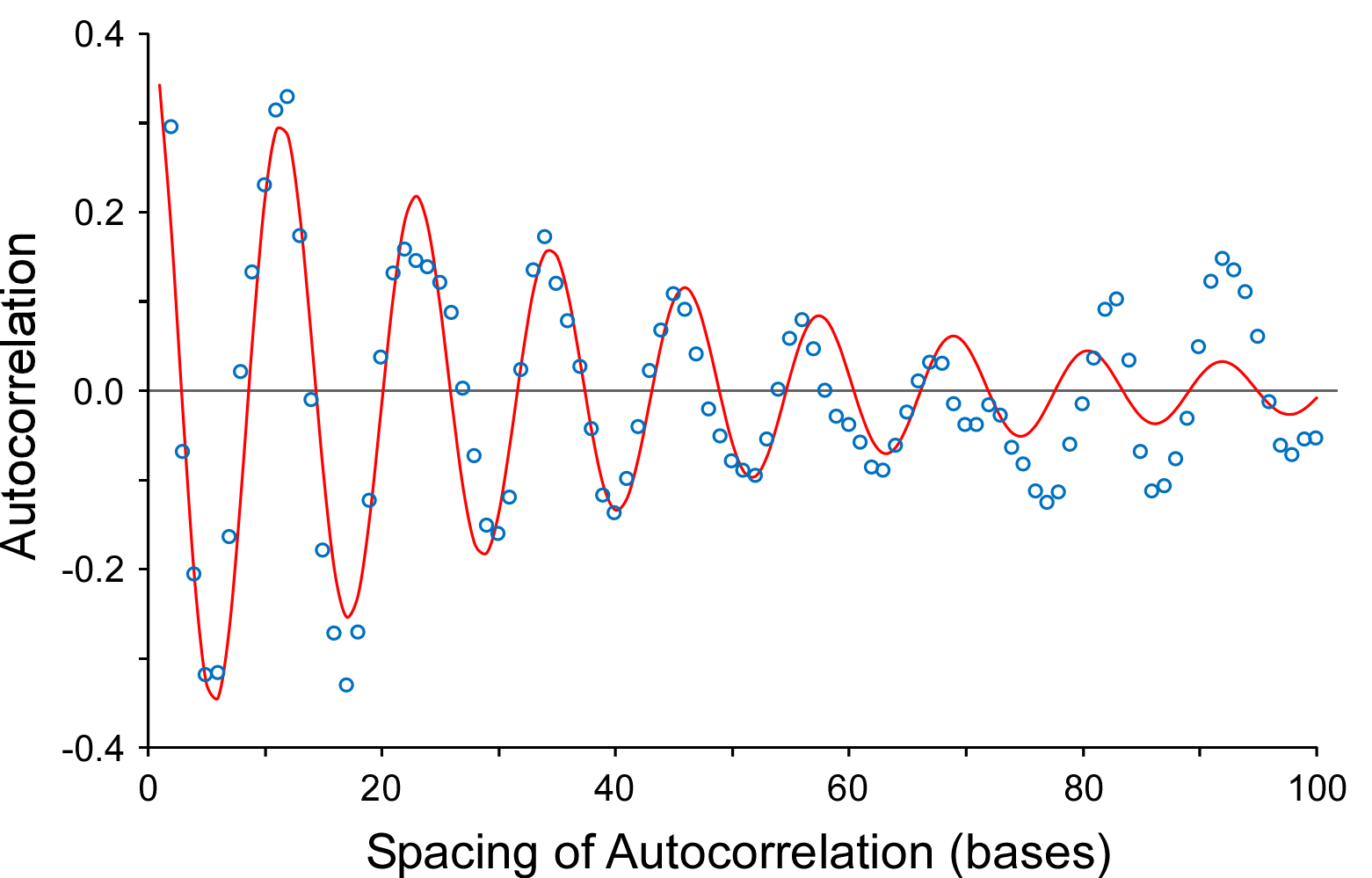

Supplement: S6 Fig — Autocorrelation of the abundances of spacings between cognate dinucleotides calculated for protein-coding genes shared between Erwinia phages Ea35-70 and vB_EamM_MadMel, removing two genes with >70% identity of their inferred proteins with bacterial genes. Spacings were measured up to 200 bp apart; autocorrelations were calculated for differences in spacings up to 100 bp (see methods). Data are shown in blue and the best fit of a damped sine curve fit to these data is shown in red. (TIF) [file pgen.1009042.s011.tif]

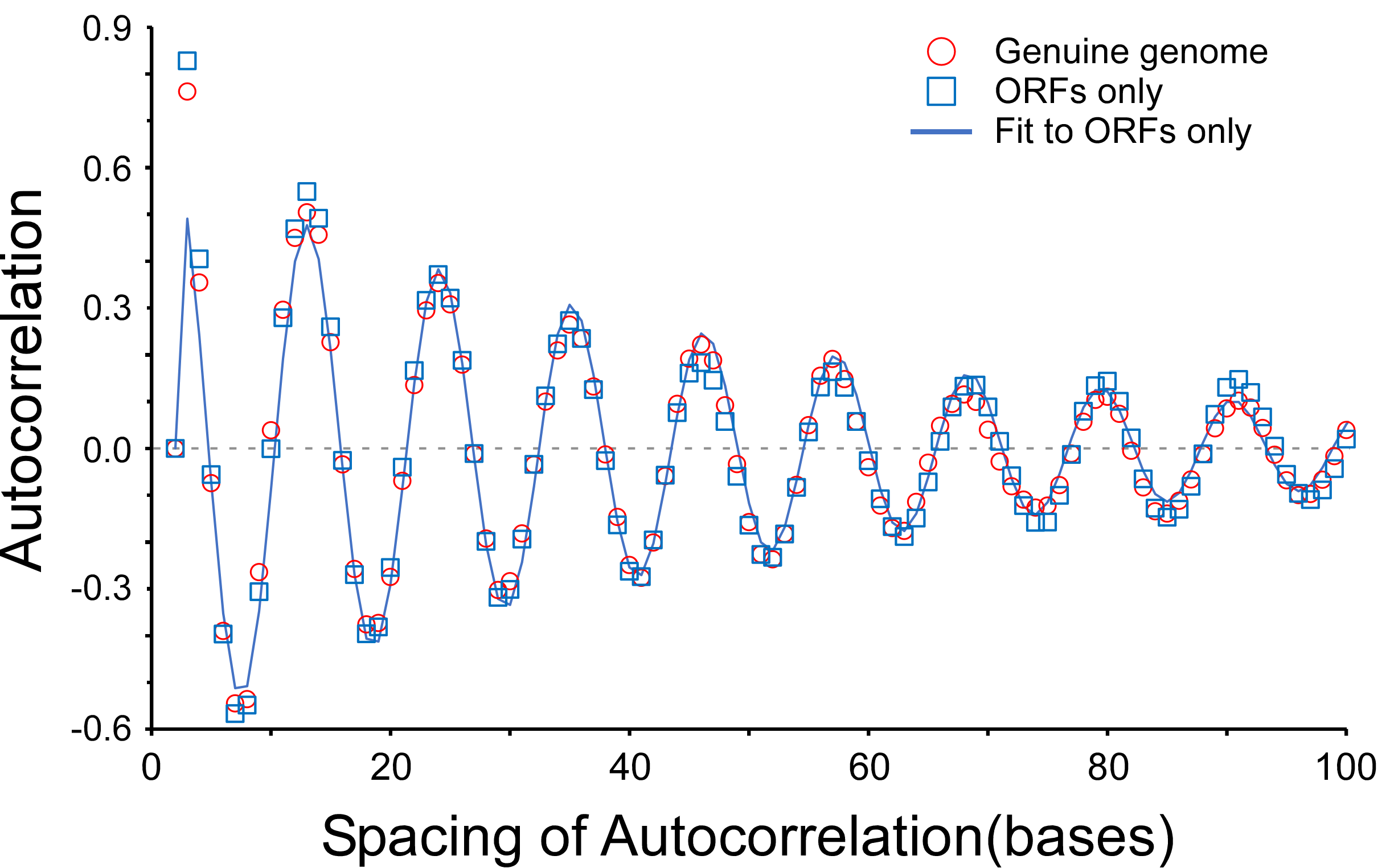

Supplement: S7 Fig — Autocorrelation of the abundances of spacings within E. coli open reading frames, with a damped sine curve fit to these data. Autocorrelation of the abundances of spacings within the entire E. coli genome (data from Fig 1) are included for comparison. (TIF) [file pgen.1009042.s012.tif]

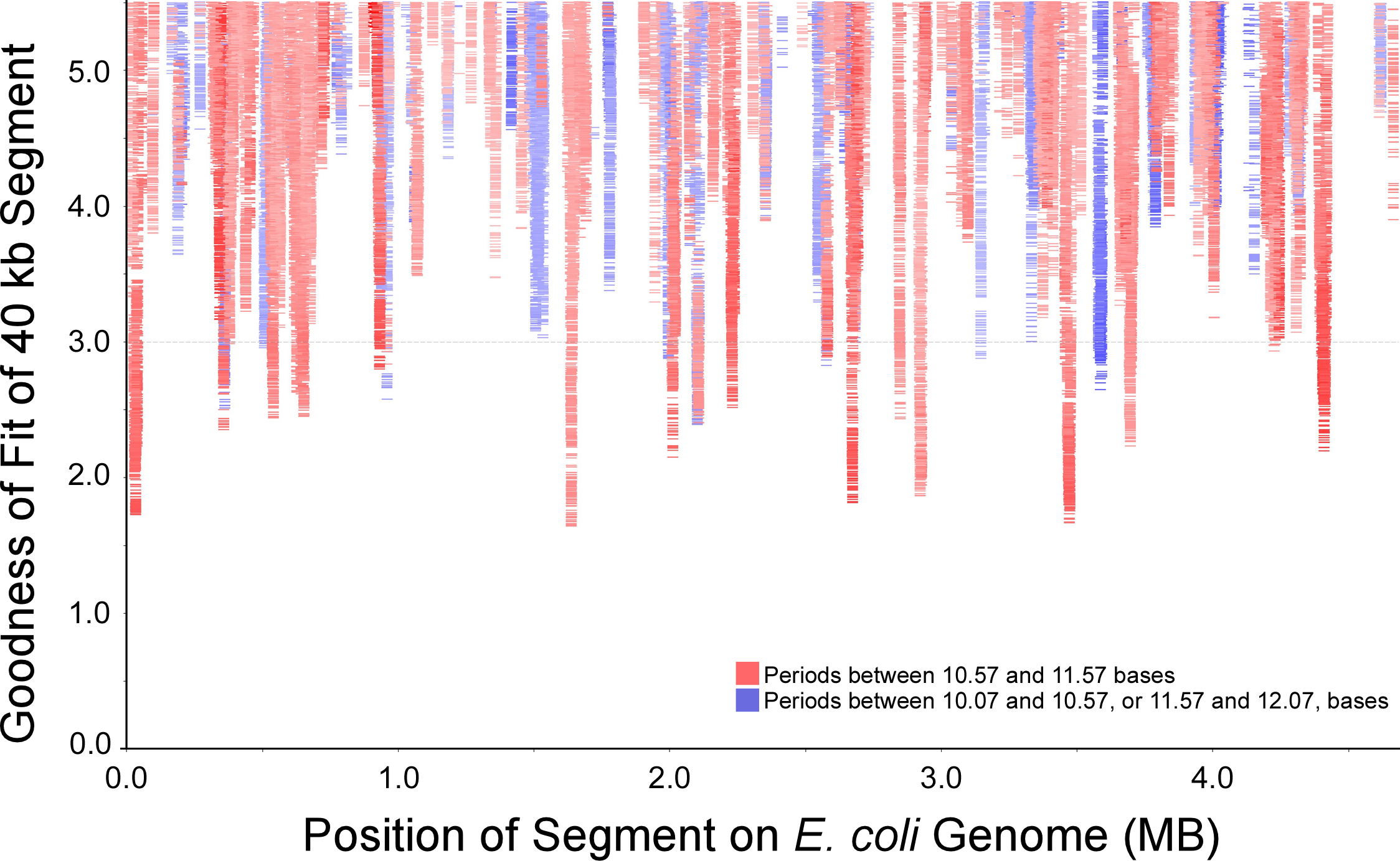

Supplement: S8 Fig — Distribution along the E. coli chromosome of 40 kb segments whose periods were estimated to be within 0.0 to 0.5 bp of the genomic period (red) and within 0.5 to 1.0 bp of the genomic period (blue) plotted as its goodness of fit to a damped sine curve. Darker shading indicates a higher amplitude (stronger periodicity). (TIF) [file pgen.1009042.s013.tif]
